# Determining Roe and Metz model parameters for simulating multireader multicase confidence-of-disease rating data based on real-data or conjectured Obuchowski–Rockette parameter estimates

**DOI:** 10.1117/1.JMI.9.4.045501

**Published:** 2022-07-08

**Authors:** Stephen L. Hillis, Brian J. Smith, Weijie Chen

**Affiliations:** a University of Iowa, Department of Radiology, Iowa City, Iowa, United States; b University of Iowa, Department of Biostatistics, Iowa City, Iowa, United States; c Office of Science and Engineering Laboratories, CDRH, FDA, Division of Imaging, Diagnostics, and Software Reliability, Silver Spring, Maryland, United States

**Keywords:** ROC curve, diagnostic radiology, Roe and Metz, Obuchowski and Rockette, simulated data

## Abstract

**Purpose:**

The most frequently used model for simulating multireader multicase (MRMC) data that emulates confidence-of-disease ratings from diagnostic imaging studies has been the Roe and Metz (RM) model, proposed by Roe and Metz in 1997 and later generalized by Hillis (2012), Abbey et al. (2013), and Gallas and Hillis (2014). A problem with these models is that it has been difficult to set model parameters such that the simulated data are similar to MRMC data encountered in practice. To remedy this situation, Hillis (2018) mapped parameters from the RM model to Obuchowski–Rockette (OR) model parameters that describe the distribution of the empirical AUC outcomes computed from the RM model simulated data. We continue that work by providing the reverse mapping, i.e., by deriving an algorithm that expresses RM parameters as functions of the OR empirical AUC distribution parameters.

**Approach:**

We solve for the corresponding RM parameters in terms of the OR parameters using numerical methods.

**Results:**

An algorithm is developed that results in, at most, one solution of RM parameter values that correspond to inputted OR parameter values. The algorithm can be implemented using an R software function. Examples are provided that illustrate the use of the algorithm. A simulation study validates the algorithm.

**Conclusions:**

The resulting algorithm makes it possible to easily determine RM model parameter values such that simulated data emulate a specific real-data study. Thus, MRMC analysis methods can be empirically tested using simulated data similar to that encountered in practice.

## 1 Introduction

For the typical diagnostic radiology study, several readers (typically radiologists) assign confidence-of-disease ratings to each case (i.e., subject) based on one or more corresponding radiologic images. The resulting data are called multireader multicase (MRMC) data. These studies are typically used to compare different imaging modalities with respect to reader performance. Often measures of reader performance are functions of the estimated receiver-operating-characteristic (ROC) curve, such as the area under the ROC curve (AUC). The Obuchowski and Rockette method (OR)^1^ is a commonly used method of analyzing reader performance outcomes which results in conclusions that generalize to both the reader and case populations.

The most frequently used model for simulating MRMC data that emulate confidence-of-disease ratings from such studies has been the model first proposed by Roe and Metz^2^ and later generalized by Hillis,^3^ Abbey,^4^ and Gallas and Hillis.^5^ We will refer to each of these models as the “Roe and Metz” (RM) model when there is no need to distinguish between them. Numerous studies have used this model for evaluating MRMC analysis and sample size methods. As discussed by Hillis,^6^ the RM model generates continuous confidence-of-disease ratings based on an underlying binormal model for each reader–test combination, with the separation between the normal and abnormal rating distributions varying across readers.

Because RM model parameters are expressed in terms of the latent rating data distribution, in contrast to MRMC analysis results that are almost always expressed in terms of parameters that describe the distribution of the reader performance outcomes, it has been difficult to set RM model parameter values such that the simulated data exhibit characteristics that are similar to MRMC data encountered in practice. To remedy this situation, Gallas and Hillis^5^ mapped the RM model parameters to variance and covariance parameters that describe the distribution of the empirical AUC outcomes computed from RM simulated data. Similarly, Hillis^6^ mapped the RM model parameters to OR parameters that describe the distribution of empirical AUC outcomes computed from RM simulated data. This paper continues that work by developing a numerical algorithm that expresses the RM parameters as functions of the empirical AUC distribution OR parameters. This result makes it easy to determine RM model parameter values such that the simulated data emulate a specific real-data study. The primary uses for the proposed algorithm are testing MRMC analysis methods and computing power estimates, using simulated MRMC data that match real data sets with respect to the empirical AUC distribution OR parameter estimates.

An outline of this paper is as follows. In Sec. 2, we discuss the original Roe and Metz model, the Hillis^3^ generalization of it, and the OR model and analysis method. In Sec. 3, we discuss the numerical OR-to-RM algorithm that maps OR parameters to RM parameters, which is derived in Appendix A for the Hillis^3^ generalization of the original RM model. In Sec. 4, we illustrate using the OR-to-RM algorithm and the previously derived RM-to-OR algorithm to simulate data emulating a real-data study, along with other examples and remarks concerning the use of the two algorithms. The paper concludes in Secs. 5 and 6.

## 2 Previous Methods

### 2.1 Roe and Metz Models: Original and Constrained Unequal-Variance

#### 2.1.1 Original RM model

Let X denote a confidence-of-disease rating assigned by a reader to a case; X is often called a decision variable (DV). The original RM simulation model proposed by Roe and Metz^2^ is a mixed four-factor (test, reader, case, and truth) ANOVA model for X with case nested within truth; test, reader, and truth crossed; test and truth treated as fixed factors; and reader and case treated as random factors. Note that we use “test” as a general term that can refer to a diagnostic test, imaging modality, or a treatment. Throughout this paper, we only consider the situation of comparing two tests.

Using the RM notation, the model is given as

$$X_{ijkt}=\mu_{t}+\tau_{it}+R_{jt}+C_{kt}+{\left(\tau R\right)}_{ijt}+{\left(\tau C\right)}_{ikt}+{\left(RC\right)}_{jkt}+{\left(\tau RC\right)}_{ijkt}+E_{ijkt},$$ (1)

where $X_{ijkt}$ denotes the confidence-of-disease rating for test i, reader j, case k of truth state t, and t=−,+, with “−” indicating a nondiseased case and “+” indicating a diseased case. Here, $\mu_{t}$ is the effect of truth state t, $\tau_{it}$ is the interaction effect of test i and truth state t, $R_{jt}$ is the interaction effect of reader j and truth state t, $C_{kt}$ is the effect of case k nested within truth state t, the multiple symbols in parentheses denote interactions, and $E_{ijkt}$ is the error term. Thus, $X_{ijkt}$ denotes the confidence-of-disease rating assigned to case k of truth state t by reader j when reading under test i. All effects are random except for $\mu_{t}$ and $\tau_{it}$. The random effects are mutually independent and normally distributed with zero means. Roe and Metz denote the corresponding variance components by $\sigma_{R}^{2}$, $\sigma_{C}^{2}$, $\sigma_{\tau R}^{2}$, $\sigma_{\tau C}^{2}$, $\sigma_{RC}^{2}$, $\sigma_{\tau RC}^{2}$, and $\sigma_{E}^{2}$. They note that $\sigma_{\tau RC}^{2}$ and $\sigma_{E}^{2}$ cannot be estimated separately for this model with no replications, as re-reading images in radiological studies is uncommon due to the cost, and hence define

$$\sigma_{\varepsilon }^{2}\equiv \sigma_{\tau RC}^{2}+\sigma_{E}^{2}.$$

Although not mentioned by Roe and Metz, the omission of test, reader, and test-by-reader effects that do not depend on truth is justified by the invariance of the ROC curve to location shifts; thus inclusion of these terms would not change the ROC curve for a given reader. Note that interactions with truth are denoted only by a t subscript in Eq. (1).

Roe and Metz constrain the sum of the error variance and variance components involving case to be equal to one:

$$\sigma_{C}^{2}+\sigma_{\tau C}^{2}+\sigma_{RC}^{2}+\sigma_{\varepsilon }^{2}=1.$$ (2)

It follows from this constraint^6^ that the fixed-reader nondiseased and diseased DV distributions have unit variances (and hence their ROC curves are symmetric about the negative 45 deg diagonal), with the fixed-reader AUCs varying across the reader population.

Without loss of generality, Roe and Metz impose the constraints

$$\mu_{-}=\tau_{1-}=\tau_{2-}=\tau_{1+}=\tau_{2+}=0,$$ (3)

which result in the same DV distributions for both tests 1 and 2. Under this constraint, it can be shown^6^ that the mean and median separation of the nondiseased and diseased DV distributions across the reader population is given by $\mu_{+}$ and the median reader-specific AUC is given by $A_{z}=\Phi (\mu_{+}/\sqrt{2})$, where Φ is the cumulative distribution function of the standard normal distribution.

#### 2.1.2 Unequal test DV distributions

Although Roe and Metz only consider simulations for equal test DV distributions for each reader, the model can be easily modified to allow for test DV distributions that differ in their median AUC values by not setting $\tau_{2+}$ to zero, that is, only the constraints

$$\mu_{-}=\tau_{1-}=\tau_{2-}=\tau_{1+}=0$$ (4)

are imposed. It follows that the median AUCs for tests 1 and 2 are equal to $A_{z}^{\left(i\right)}=\Phi (\delta_{i}/\sqrt{2})$, i=1,2, respectively, where

$$\delta_{i}=\mu_{+}+\tau_{i+}\;i=1,2$$ (5)

are the mean and median separations of the nondiseased and diseased DV distributions for tests 1 and 2, respectively, across the reader population. From constraints Eq. (4), it follows that $\delta_{1}=\mu_{+}$ for test 1 and $\delta_{2}=\mu_{+}+\tau_{2+}$ for test 2. To insure that $A_{z}^{\left(i\right)}\geq .5$, we assume

$$\delta_{i}\geq 0,\;i=1,2.$$ (6)

Note that the RM model that allows for test-dependent AUCs is completely defined by seven parameters:

$$\delta_{1},\delta_{2},\sigma_{R}^{2},\sigma_{\tau R}^{2},\sigma_{C}^{2},\sigma_{\tau C}^{2},\;\text{and}\;\sigma_{RC}^{2}.$$ (7)

Note that $\sigma_{\varepsilon }^{2}$ can be computed using Eqs. (2) and (7).

#### 2.1.3 Constrained unequal-variance RM model (RMH model)

In practice, estimated binormal-model nondiseased and diseased distribution variances for a reader-test combination are often different, with diseased subjects typically having more variable test results. Thus to better emulate real data, Hillis^3^ modified the original RM model by allowing variance components involving cases to depend on truth, with variance components involving diseased cases set equal to those involving nondiseased cases multiplied by the factor $1/b^{2}$, b>0. Specifically, the model is given by Eq. (1) with variance components (using an obvious notation) denoted by $\sigma_{R}^{2}$, $\sigma_{\tau R}^{2}$, $\sigma_{C(-)}^{2}$, $\sigma_{\tau C(-)}^{2}$, $\sigma_{RC(-)}^{2}$, $\sigma_{\varepsilon (-)}^{2}$, $\sigma_{C(+)}^{2}$, $\sigma_{\tau C(+)}^{2}$, $\sigma_{RC(+)}^{2}$, and $\sigma_{\varepsilon (+)}^{2}$, with $\sigma_{C(+)}^{2}=b^{-2}\sigma_{C(-)}^{2}$, $\sigma_{\tau C(+)}^{2}=b^{-2}\sigma_{\tau C(-)}^{2}$, $\sigma_{RC(+)}^{2}=b^{-2}\sigma_{RC(-)}^{2}$, $\sigma_{\varepsilon (+)}^{2}=b^{-2}\sigma_{\varepsilon (-)}^{2}$. Similar to Eq. (2), the constraint

$$\sigma_{C(-)}^{2}+\sigma_{\tau C(-)}^{2}+\sigma_{RC(-)}^{2}+\sigma_{\varepsilon (-)}^{2}=1$$ (8)

is imposed. It follows that

$$\sigma_{C(+)}^{2}+\sigma_{\tau C(+)}^{2}+\sigma_{RC(+)}^{2}+\sigma_{\varepsilon (+)}^{2}=b^{-2}.$$

Constraint Eq. (6) is also imposed. We will refer to this model as the constrained unequal-variance RM model or simply as the RMH model, with the “H” in RMH indicating that it is the generalization of the original RM model proposed by Hillis.^3^

Similar to the original RM model,^2^ imposing constraint Eq. (3) results in the null model with $A_{z}^{\left(1\right)}=A_{z}^{\left(2\right)}=\Phi (\mu_{+}/\sqrt{1+b^{-2}})$, and imposing constraint Eq. (4) results in the nonnull model with

$$A_{z}^{\left(i\right)}=\Phi ((\mu_{+}+\tau_{i+})/\sqrt{1+b^{-2}})\;=\Phi (\delta_{i}/\sqrt{1+b^{-2}}),\;i=1,2,$$

where again $A_{z}^{\left(i\right)}$ denotes the median AUC across the reader population for test i, $\delta_{i}$ is defined by Eq. (5), and $\delta_{i}$ is the mean and median DV separation for test i across readers.

The algorithm discussed in this paper will be for the RMH model, which includes the original RM model^2^ as a special case when b is set equal to 1. Note that the RMH model that allows for test-dependent AUCs is completely defined by the eight linearly independent parameters $b,\delta_{1},\delta_{2},\sigma_{R}^{2},\sigma_{\tau R}^{2},\sigma_{C(-)}^{2},\sigma_{\tau C(-)}^{2}$, and $\sigma_{RC(-)}^{2}$. We let $\beta_{\mathrm{RMH}}$ denote the vector of these parameters:

$$\beta_{\mathrm{RMH}}=(b,\delta_{1},\delta_{2},\sigma_{R}^{2},\sigma_{\tau R}^{2},\sigma_{C(-)}^{2},\sigma_{\tau C(-)}^{2},\sigma_{RC(-)}^{2}).$$ (9)

### 2.2 Obuchowski–Rockette Model

Obuchowski and Rockette^1^ proposed a test × reader factorial ANOVA model for the AUC estimates, but unlike a conventional ANOVA model, the errors are assumed to be correlated to account for correlation due to each reader evaluating the same cases. Their model, which we refer to as the OR model, is given as

θ^ij=μOR+τi:OR+Rj:OR+(τR)ij:OR+εij:OR, (10)

where $\mu_{\mathrm{OR}}$ is the intercept term, $\tau_{i:\mathrm{OR}}$ denotes the fixed effect of test i, $R_{j:\mathrm{OR}}$ denotes the random effect of reader j, ${\left(\tau R\right)}_{ij:\mathrm{OR}}$ denotes the random test × reader interaction, and $\varepsilon_{ij:\mathrm{OR}}$ is the error term. The $R_{j:\mathrm{OR}}$ and ${\left(\tau R\right)}_{ij:\mathrm{OR}}$ are assumed to be mutually independent and normally distributed with zero means and respective variances $\sigma_{R:\mathrm{OR}}^{2}$ and $\sigma_{TR:\mathrm{OR}}^{2}$. (OR in the subscripts is to distinguish OR effects and variance components from similarly notated RMH-model quantities.) The $\varepsilon_{ij:\mathrm{OR}}$ are assumed to be normally distributed with mean zero and variance $\sigma_{\varepsilon :\mathrm{OR}}^{2}$ and are assumed uncorrelated with the $R_{j:\mathrm{OR}}$ and ${\left(\tau R\right)}_{ij:\mathrm{OR}}$. Three possible error covariances are assumed:

$$\mathrm{Cov}(\varepsilon_{ij:\mathrm{OR}},\varepsilon_{i^{\prime }j^{\prime }:\mathrm{OR}})=\{\begin{array}{ll} {\mathrm{Cov}}_{1} & i\neq i^{\prime },j=j^{\prime }(\text{different test},\text{same reader})\\ {\mathrm{Cov}}_{2} & i=i^{\prime },j\neq j^{\prime }(\text{same test},\text{different reader})\\ {\mathrm{Cov}}_{3} & i\neq i^{\prime },j\neq j^{\prime }(\text{different test},\text{different reader}) \end{array}.$$ (11)

The OR model assumes^7^

$${\mathrm{Cov}}_{1}\geq {\mathrm{Cov}}_{3},\;{\mathrm{Cov}}_{2}\geq {\mathrm{Cov}}_{3},\;{\mathrm{Cov}}_{3}\geq 0.$$ (12)

These error variance–covariance parameters are typically estimated by averaging corresponding conditional-on-readers estimates computed using the jackknife,^8^^–^^10^ bootstrap,^10^^,^^11^ the method proposed by DeLong et al.^12^ (for empirical AUC estimates), or the method proposed by Metz et al.^13^ based on the semiparametric binormal ROC model. These four estimation methods are consistent but are not unbiased. An unbiased error covariance estimation method (unbiased method) was recently proposed by Hillis^6^^,^^14^ for use when empirical AUC is the outcome. This method utilizes the unbiased fixed-reader method discussed by Gallas [Ref. 15, p 362] for estimating the error variance, and extensions of it for estimating the error covariances. This method results in unbiased OR parameter estimates when data are generated from the RMH model.^6^ OR analysis using this method is included in the freely available R software package MRMCaov.^16^

The $\varepsilon_{ij:\mathrm{OR}}$ can be interpreted as AUC measurement error attributable to the random selection of cases and within-reader variability that describes how a fixed reader interprets the same image in different ways on different occasions. The OR model can alternatively be described with population correlations $r_{i}={\mathrm{Cov}}_{i}/\sigma_{\varepsilon :\mathrm{OR}}^{2}$ replacing corresponding ${\mathrm{Cov}}_{i}$.

Defining

$$\mu_{1:\mathrm{OR}}=\mu_{\mathrm{OR}}+\tau_{1:\mathrm{OR}},\;\mu_{2:\mathrm{OR}}=\mu_{\mathrm{OR}}+\tau_{2:\mathrm{OR}},$$

the OR model for two tests, similar to the RMH model, is defined by eight linearly independent parameters:

$$\mu_{1:\mathrm{OR}},\mu_{2:\mathrm{OR}},\sigma_{R:\mathrm{OR}}^{2},\sigma_{TR:\mathrm{OR}}^{2},\sigma_{\varepsilon :\mathrm{OR}}^{2},{\mathrm{Cov}}_{1},{\mathrm{Cov}}_{2},\;\text{and}\;{\mathrm{Cov}}_{3},$$ (13)

or equivalently, by

$$\mu_{1:\mathrm{OR}},\mu_{2:\mathrm{OR}},\sigma_{R:\mathrm{OR}}^{2},\sigma_{TR:\mathrm{OR}}^{2},\sigma_{\varepsilon :\mathrm{OR}}^{2},r_{1},r_{2}\;\text{and}\;r_{3}.$$ (14)

We let $\beta_{\mathrm{OR}}$ denote the vector of these parameters:

$$\beta_{\mathrm{OR}}=(\mu_{1:\mathrm{OR}},\mu_{2:\mathrm{OR}},\sigma_{R:\mathrm{OR}}^{2},\sigma_{TR:\mathrm{OR}}^{2},\sigma_{\varepsilon :\mathrm{OR}}^{2},r_{1},r_{2},r_{3}).$$ (15)

Note that when the outcome is the empirical AUC that $\mu_{1:\mathrm{OR}}$ and $\mu_{2:\mathrm{OR}}$ are the test 1 and test 2 expected values for the empirical AUC estimates across readers and cases.

## 3 Proposed Methods

### 3.1 OR-to-RMH Algorithm for Estimating RMH Parameter Values When the Goal Is to Emulate a Real-Data MRMC Study

The RMH-to-OR mapping, previously derived by Hillis,^6^ and the new OR-to-RMH algorithm that maps OR parameters to RMH parameters and its development are provided in Tables 6 and 10, respectively, in Appendix A.

In this section, we discuss the main points of the OR-to-RMH algorithm when the goal is to emulate data from a real study with the RMH model; i.e., to determine RMH parameter values such that the expected values of the OR parameter estimates from the simulated MRMC samples are described by the $\beta_{\mathrm{OR}}$ vector Eq. (15), estimated from a real study.

The $\beta_{\mathrm{OR}}$ vector Eq. (15) implicitly provides information about the shape of the underlying ROC curve through the value of $\sigma_{\varepsilon :\mathrm{OR}}^{2}$, which is a function of the RMH b parameter in the RMH-to-OR mapping. The method used for estimating the RMH b parameter for the OR-to-RMH algorithm is called the b_method. To estimate a $\beta_{\mathrm{RMH}}$ vector Eq. (9) that maps to a particular $\beta_{\mathrm{OR}}$ vector Eq. (15), the algorithm requires use of the option b_method = unspecified, which we assume throughout this section. Two other options for b_method and the situations where they are useful will be discussed in Sec. 3.2.

#### 3.1.1 Overview of OR-to-RMH algorithm

Table 6 in Appendix A gives the previously derived analytical RMH-to-OR mapping formulas.^6^ Mathematically, we describe this transformation by the function f that maps the RMH parameter vector and the case samples sizes that will be used for the simulations to the resulting OR parameter vector:

$$f(\beta_{\mathrm{RMH}},n_{0},n_{1})=\beta_{\mathrm{OR}}.$$ (16)

This function is analytical and thus does not require a numerical algorithm.

The OR-to-RMH algorithm requires inputted values for $\beta_{\mathrm{OR}},n_{0},n_{1}$, and b_method, where $\beta_{\mathrm{OR}}$ is given by Eq. (15) and $n_{0}$ and $n_{1}$ are the corresponding real-study nondiseased and diseased case sizes. To derive the OR-to-RMH algorithm, we first assume that there exists an RMH parameter vector $\beta_{\mathrm{RMH}}$ corresponding to $\beta_{\mathrm{OR}}$ such that Eq. (16) is true. We then express the OR parameters in terms of the RMH parameters and solve for the RMH parameters using numerical methods (see Appendix A for details.)

It is possible that there are several $\beta_{\mathrm{RMH}}$ vectors satisfying Eq. (16), in which case the corresponding $\beta_{\mathrm{RMH}}$ vectors will differ only in their b values, as discussed in Appendix A. It is also possible that there is no $\beta_{\mathrm{RMH}}$ vector that satisfies Eq. (16). To force the OR-to-RMH algorithm to produce, at most, only one output, the $\beta_{\mathrm{RMH}}$ vector with b closest to 1 with 0.01≤b≤1 is chosen; if no corresponding $\beta_{\mathrm{RMH}}$ vector has 0.01≤b≤1, then the corresponding $\beta_{\mathrm{RMH}}$ vector with b closest to 1 with 1<b≤4 is chosen. If there are no corresponding $\beta_{\mathrm{RMH}}$ solution vectors with 0.01≤b≤4, the algorithm does not return a solution for $\beta_{\mathrm{RMH}}$; see Sec. 3.1.3 for what to do when this happens.

Let $g_{1}$ denote the function defined by the OR-to-RMH algorithm, with b_method = unspecified, that maps $\beta_{\mathrm{OR}}$ to a solution for $\beta_{\mathrm{RMH}}$, denoted by $\beta_{\mathrm{RMH};\text{solution}}$; i.e.,

$$g_{1}(\beta_{\mathrm{OR}},n_{0},n_{1})=\beta_{\mathrm{RMH};\text{solution}}.$$ (17)

Ideally, $\beta_{\mathrm{RMH};\mathrm{solution}}$ will be such that the RMH-to-OR mapping will return the original OR parameter, i.e.,

$$f(\beta_{\mathrm{RMH};\text{solution}},n_{0},n_{1})=\beta_{\mathrm{OR}}.$$ (18)

However, it is possible for the OR-to-RMH algorithm to return a solution such that Eq. (18) holds only approximately, i.e.,

$$f(\beta_{\mathrm{RMH};\text{solution}},n_{0},n_{1})\approx \beta_{\mathrm{OR}}.$$ (19)

The approximation results because of constraints on the RMH parameters that are imposed by the algorithm, as discussed in Appendix A and given in Eq. (23) in Table 7. For example, if the inputted value of $r_{3}$ exceeds that of $r_{2}$ then the solution $\beta_{\mathrm{RMH};\text{solution}}$ will be such that $r_{2}\geq r_{3}$ in $f(\beta_{\mathrm{RMH};\text{solution}},n_{0},n_{1})$.

##### Rationale for the b limits

The lower and upper limits for b of 0.01 and 4 are chosen because b values outside these limits are not realistic for most real data sets. In most situations, a meaningful DV should be an increasing transformation of the likelihood ratio (likelihood of being diseased divided by likelihood of not being diseased).^17^ A DV having this property and its corresponding ROC curve are said to be proper; otherwise they are said to be improper [Ref. 18, pp. 19, 37]. A proper ROC curve is concave (down) and never crosses the chance line.^17^ It follows that an ROC curve that has “hooks” and crosses the chance line is improper. Pan and Metz^19^ note that hooks for fitted binormal ROC curves do not appear when fitting curves to reliable data sets, which strongly suggests that the true underlying ROC curves do not show such hooks for real-data studies. Thus, we have limited the underlying ROC curves to have b values between 0.01 and 4.0 since for typical AUC values (≤0.95) it can be shown that ROC curves with b values outside of these boundaries have noticeable hooks.

**Fig. 1:**
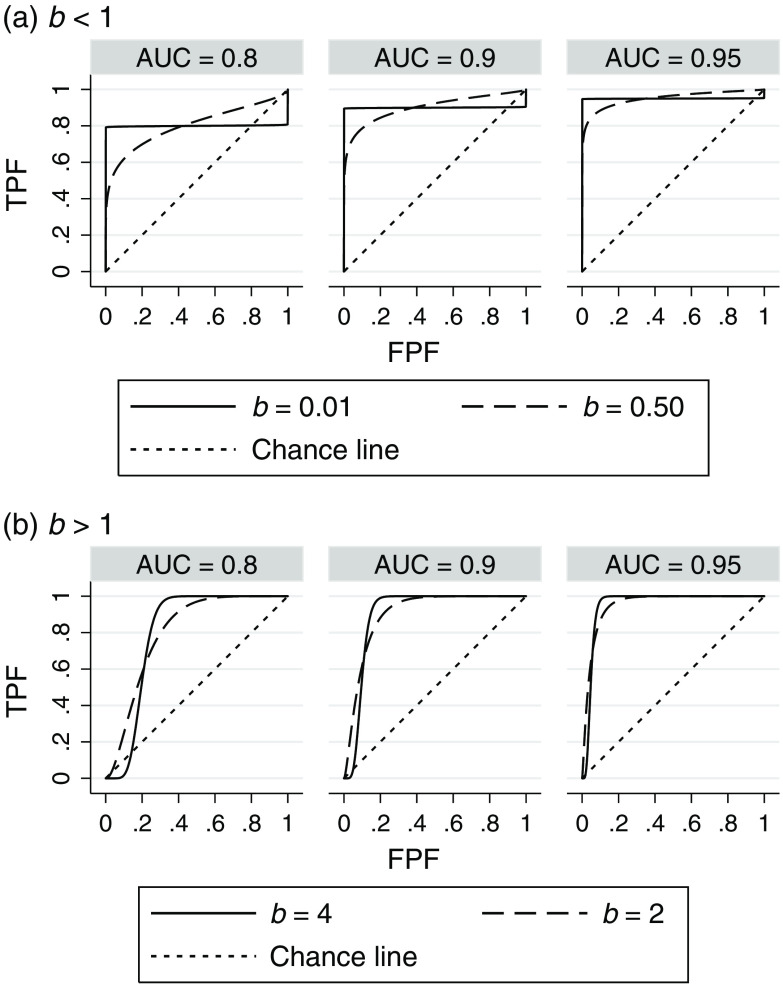
ROC curves as a function of AUC and b. TPF, true positive fraction (or sensitivity); FPF, false positive fraction (or 1 – specificity).

For example, Fig. 1 shows ROC curves with AUCs of 0.8, 0.9, 0.95 for values of b=0.01,0.5 [Fig. 1(a)] and b=2,4 [Fig. 1(b)]. We see that the ROC curves for the extreme cases of b=0.01 [Fig. 1(a)] and b=4 [Fig. 1(b)] are noticeably improper because they have hooks in the upper right and lower left corner, respectively, with the ROC curves below the chance line in those regions. Although not shown, the improperness becomes more noticeable as b decreases below 0.01 or increases above 4.0, or as the AUC decreases below 0.8. The ROC curves were computed using the equation $\mathrm{TPF}=\Phi (a+b\Phi^{-1}\mathrm{FPF})$, with $a=b\Phi^{-1}(\mathrm{AUC})\sqrt{1+(\frac{1}{b}{)}^{2}}$ and TPF and FPF denoting the true positive fraction (sensitivity) and false positive fraction (1 − specificity), respectively. (The expression for a results from the conventional binormal ROC relationships $\mu =\Phi^{-1}(\mathrm{AUC})\sqrt{1+{\left(\frac{1}{b}\right)}^{2}}$ and μ=a/b).

##### Simulation of data to emulate a real-data study

**Fig. 2:**
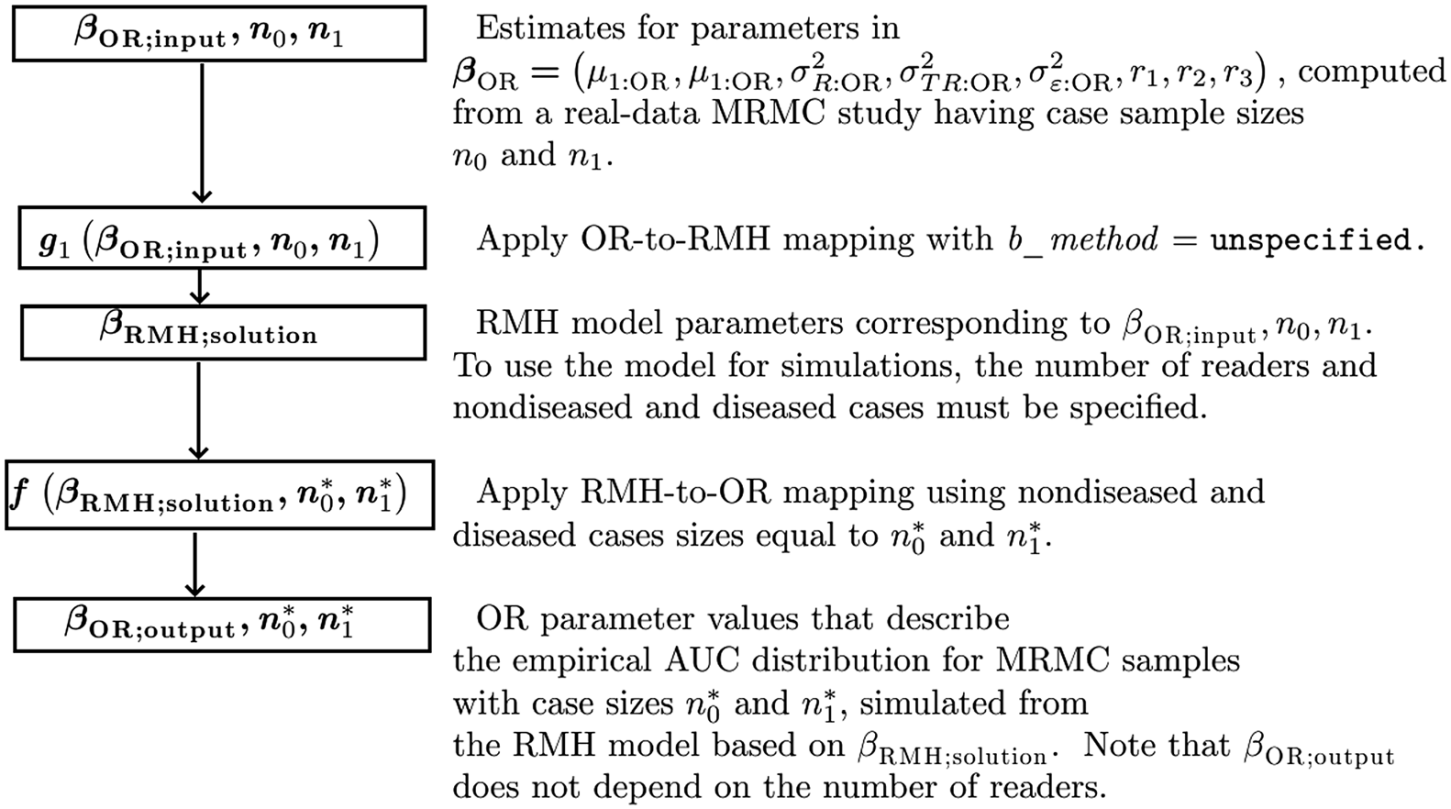
Flowchart illustrating the use of the OR-to-RMH and RMH-to-OR algorithms to simulate MRMC data that emulate a real-data study.

Figure 2 summarizes how the OR-to-RMH and RMH-to-OR algorithms can be used to simulate data that emulate a real-data study. The OR-to-RMH algorithm (with b_method = unspecified) is applied to OR estimates ($\beta_{\mathrm{OR};\text{input}}$) obtained from a real-data study, resulting in the corresponding RMH model. This model is then used for generating MRMC samples for any specified number of readers and cases, with $n_{0}^{*}$ and $n_{1}^{*}$ denoting the case numbers for the simulations and $n_{0}$ and $n_{1}$ denoting the case numbers for the original real-data study. The distribution of the empirical AUCs for the simulated data is described by $\beta_{\mathrm{OR};\text{output}}$. We recommend always checking how closely the simulated data emulate the study data by comparing $\beta_{\mathrm{OR};\text{input}}$ and $\beta_{\mathrm{OR};\text{output}}$ when the simulation model generates samples with the same case sizes as the original study, i.e., with $n_{0}^{*}=n_{0}$ and $n_{1}^{*}=n_{1}$.

#### 3.1.2 Should the simulated ROC curves resemble the original study ROC curves?

**Fig. 3:**
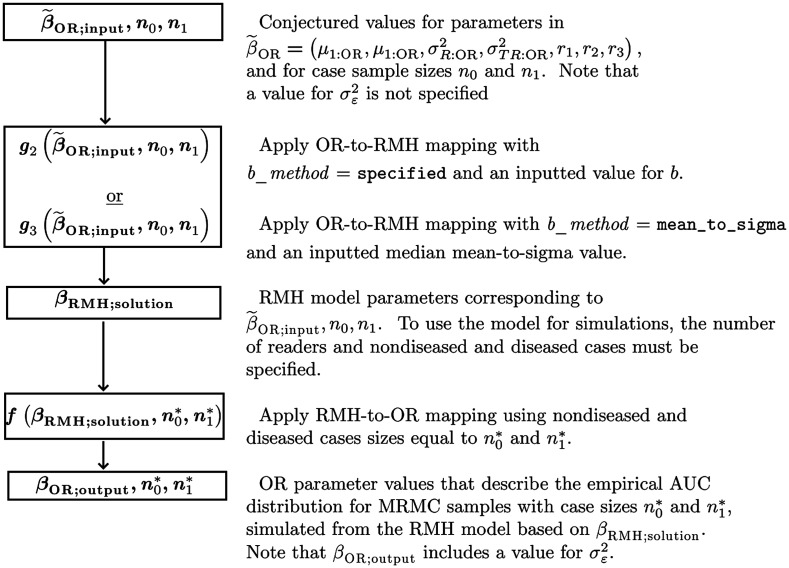
Flowchart illustrating the use of the OR-to-RMH and RMH-to-OR algorithms to simulate MRMC data that emulates OR AUCs, reader variance components, and OR correlations, but not $\sigma_{\varepsilon :\mathrm{OR}}^{2}$.

We emphasize that even when simulating data using an RMH model such that $\beta_{\mathrm{OR};\text{output}}=\beta_{\mathrm{OR}:\text{input}}$ in Fig. 3, we do not claim that the resulting empirical ROC curves will be visually similar to those estimated from a real-data study. Rather, we only claim that the expected values of the OR parameter estimates for the simulated data will be the same as those computed from the original real-data study, given by Eq. (13). (Note that Eq. (13) contains the error covariances rather than the error correlations.) However, because of the robustness of the binormal model assumption for fitting ROC curves to real data,^20^^–^^22^ we typically expect there will be some resemblance, although the degree of resemblance will be limited by the RMH model having only eight parameters. In particular, we note that the RMH model requires each reader’s ROC curve to have the same b value, which will determine the shape of the ROC curve for a given reader AUC value; this result follows from the one-to-one correspondence between (b,AUC) and (a,b), with $a=b\Phi (\mathrm{AUC})\sqrt{1+(\frac{1}{b}{)}^{2}}$, as mentioned in Sec. 3.1.1.

#### 3.1.3 Reasons for neither an exact nor approximate solution

##### OR-to-RMH algorithm does not work because there is not a solution for b

For given values of the RMH parameters $\delta_{1},\delta_{2},\sigma_{R}^{2}$, and $\sigma_{TR}^{2}$ (computed in steps 1 to 3 of the OR-to-RMH algorithm in Table 10), the value of b (computed in step 4) determines the value of $\sigma_{\varepsilon ;\mathrm{OR}}^{2}.$ It can happen that the algorithm does not produce a solution for b, either because no solution exists, or the solution is <0.01 or >4.0 that will yield the input value for $\sigma_{\varepsilon :\mathrm{OR}}^{2}$ for the values of $\delta_{1},\delta_{2},\sigma_{R}^{2}$, and $\sigma_{TR}^{2}$ that have been computed by the algorithm in previous steps. When this occurs, one can choose to use one of the other two methods for estimating b, as discussed in Sec. 3.2.

##### OR-to-RMH algorithm does not work because there is not a solution for an RMH parameter other than b

When required, the algorithm imposes the constraints in Table 7(b) by altering somewhat the inputted OR parameter values, which can lead to an approximate solution as given by Eq. (19). However, when other constraints, which are implied by the RMH-to-OR mapping in Table 6, do not hold, the result is a missing value for the particular RMH parameter and for all other RMH parameters requiring it for their computation. For example, from the equations in Tables 8 and 9, it can be shown that there is an upper limit for $\sigma_{R:\mathrm{OR}}^{2}$, which is a function of the values of the inputted values for $\mu_{1:\mathrm{OR}}$ and $\mu_{2:\mathrm{OR}}$. Similarly, it can be shown that there are upper limits for $\sigma_{TR:\mathrm{OR}}^{2},r_{1},r_{2}$, and $r_{3}$, which are functions of parameters computed in previous steps. When one of these values exceeds its upper limit, the algorithm does not yield a solution.

This problem is more likely to happen when inputted values for $\beta_{\mathrm{OR}}$ are conjectured than when they are estimates from a real-data study. If this problem occurs, we first recommend that the inputted values be checked for entry errors. If there are none, then we suggest inputting a different (typically smaller) value for the OR parameter corresponding to the RMH parameter, which cannot be estimated. See Appendix A and Table 5 for more details and Sec. 4.3.7 for examples illustrating this problem.

### 3.2 OR-to-RMH Algorithm for Estimating RMH Parameter Values When the Goal Is to Emulate AUCs, OR Correlations and Variance Components, But Not $\sigma_{\varepsilon :\mathrm{OR}}^{2}$

As discussed by Hillis,^6^^,^^23^ the OR parameters $\mu_{1:\mathrm{OR}},\mu_{2:\mathrm{OR}},\sigma_{R:\mathrm{OR}}^{2}$, and $\sigma_{TR:\mathrm{OR}}^{2}$ have meaningful interpretations that do not depend on sample size, and $r_{1}$, $r_{2}$, and $r_{3}$ have meaningful interpretations that remain approximately (but not exactly) constant as the sample sizes change. On the other hand, $\sigma_{\varepsilon :\mathrm{OR}}^{2}$ varies with the sample sizes. In this section, we discuss two approaches for determining RMH parameters that result in simulated MRMC data for which the empirical AUC distribution matches conjectured values of the parameters in

$${\overset{˜}{\beta }}_{\mathrm{OR}}=(\mu_{1:\mathrm{OR}},\mu_{2:\mathrm{OR}},\sigma_{R:\mathrm{OR}}^{2},\sigma_{TR:\mathrm{OR}}^{2},r_{1},r_{2},r_{3}).$$

Note that ${\tilde{\beta }}_{\mathrm{OR}}$ is the same as $\beta_{\mathrm{OR}}$ but without $\sigma_{\varepsilon ;\mathrm{OR}}^{2}$. The value of $\sigma_{\varepsilon :\mathrm{OR}}^{2}$ for the simulated data will be determined by the sample sizes and the RMH parameters.

These approaches are useful when one is primarily interested in simulating data that match an OR correlation and variance component structure and a real-data value of $\sigma_{\varepsilon :\mathrm{OR}}^{2}$ is not available. They also are useful when real-data estimates for $\beta_{\mathrm{OR}}$ are available but there is no solution for b using the OR-to-RMH algorithm with b_method = unspecified.

#### 3.2.1 Overview

The two approaches are similar to that described in Sec. 3.1, except that estimation of b does not depend on an inputted value for $\sigma_{\varepsilon :\mathrm{OR}}^{2}$. Instead, b is either (1) explicitly specified using b_method = specified and setting the value of the input variable b_input equal to the desired value for b; or (2) computed so as to result in a median specified mean-to-sigma ratio across readers, using b_method = mean_to_sigma and setting the value of the input variable mean_sig_input equal to the desired mean-to-sigma ratio.

Use of the OR-to-RMH and RMH-to-OR algorithms to simulate data using these two approaches is summarized in Fig. 3. Figure 3 is similar to Fig. 2 with these differences: (1) No input value for $\sigma_{\varepsilon :\mathrm{OR}}^{2}$ is included because the input values are for ${\overset{˜}{\beta }}_{\mathrm{OR}}$ instead of for $\beta_{\mathrm{OR}}$. (2) For the OR-to-RMH algorithm, the $g_{2}$ or $g_{3}$ function (as defined below) is used in the place of the $g_{1}$ function. Note that the outputted OR parameter values include a value for $\sigma_{\varepsilon :\mathrm{OR}}^{2}$.

##### Approach 1: b_method = specified

With this approach, the value of b is specified. For example, the parameter values for the original^2^ RM model can be determined by setting b_input=1.

Let $g_{2}$ denote the function defined by the OR-to-RMH algorithm, with b_method = specified, that maps ${\tilde{\beta }}_{\mathrm{OR}}$ and an inputted value of b to a solution for $\beta_{\mathrm{RMH}}$, denoted by $\beta_{\mathrm{RMH};\text{solution}}$; i.e.,

$$g_{2}({\overset{˜}{\beta }}_{\mathrm{OR}},n_{0},n_{1},b\_\text{input})=\beta_{\mathrm{RMH};\text{solution}}.$$ (20)

Again, ideally $\beta_{\mathrm{RMH};\text{solution}}$ will be such that $f(\beta_{\mathrm{RMH};\text{solution}},n_{0},n_{1})={\overset{˜}{\beta }}_{\mathrm{OR}}$. However, similar to using b_method = unspecified, it is possible for the OR-to-RMH algorithm to return a solution such that $f(\beta_{\mathrm{RMH};\text{solution}},n_{0},n_{1})\approx {\overset{˜}{\beta }}_{\mathrm{OR}}$ because of constraints on the RMH parameters Eq. (23) in Table 7 that are imposed by the algorithm.

##### Approach 2: b_method = mean_to_sigma

Recall from Sec. 3.1.3 that when b_method = unspecified is used, the value of b (based on the computed values of the RMH parameters $\delta_{1},\delta_{2},\sigma_{R}^{2}$ and $\sigma_{TR}^{2}$) is determined such that $\sigma_{\varepsilon :\mathrm{OR}}^{2}$ for the simulated data will match the inputted value for $\sigma_{\varepsilon :\mathrm{OR}}^{2}$. In contrast, when b_method = mean_to_sigma is used, the user specifies a desired median mean-to-sigma value (see discussion of the mean-to-sigma measure below) across readers for the test corresponding to the minimum of the inputted $\mu_{1:\mathrm{OR}}$ and $\mu_{2:\mathrm{OR}}$ values.

Let $g_{3}$ denote the function defined by the OR-to-RMH algorithm with b_method = mean_to_sigma that maps ${\tilde{\beta }}_{\mathrm{OR}}$ and an inputted value of the mean-to-sigma ratio, denoted by q, to a solution for $\beta_{\mathrm{RMH}}$:

$$g_{3}({\overset{˜}{\beta }}_{\mathrm{OR}},n_{0},n_{1},q)=\beta_{\mathrm{RMH};\text{solution}}.$$ (21)

As was the case for the other two b estimation methods, ideally, $f(\beta_{\mathrm{RHM};\text{solution}},n_{0},n_{1})={\overset{˜}{\beta }}_{\mathrm{OR}}$, but it is possible for this relationship to hold only approximately because of constraints on the RMH parameters.

#### 3.2.2 Mean-to-sigma ratio

The mean-to-sigma ratio, denoted by q, is defined as the difference of the latent diseased and nondiseased DV means divided by the difference of their standard deviations. The mean-to-sigma ratio was first introduced by Swets,^24^ who noticed that it seemed to be approximately constant for a variety of experiments. Some support for this conclusion was provided by later analyses.^22^^,^^25^^,^^26^ For example, Green and Swets^26^ note that q≈4 is typical for many studies.

As discussed by Hillis and Berbaum,^27^ q can be used as a measure of improperness for a binormal ROC curve; specifically, it indicates that the ROC curve crosses the chance line at fpf=Φ(q), where fpf is the false positive fraction. They point out that it follows that an absolute value <2 indicates a noticeably improper binormal curve and an absolute value of infinity indicates a symmetric curve (b=1).

For the RMH model, the mean-to-sigma ratio varies across readers. To avoid simulating data based on visibly improper binormal curves, we suggest that the probability of a reader’s true ROC curve being noticeably improper be small for each test, e.g., <0.025. This probability can be computed as a function of the RMH parameters, as discussed in Appendix B.1.

## 4 Results and Examples

### 4.1 R language Functions

Two functions written in the R statistical software language that perform the OR-to-RMH and RMH-to-OR mappings are available within the freely available **MRMCaov** **R** package,^16^ which can be downloaded from the Github repository: https://github.com/brian-j-smith/MRMCaov. The function *OR_to_RMH* transforms OR parameters to RMH parameters using the numerical algorithm described in Table 10, and the function *RMH_to_OR* performs the analytical RMH-to-OR transformation, described in Table 6.

### 4.2 Example: Using the Algorithms to Simulate Data Emulating a Real-Data Study

#### 4.2.1 Approach

In this section, we illustrate the use of the algorithms to simulate data that emulate data provided by Carolyn Van Dyke (VanDyke),^28^ which we have used for examples in previous papers,^29^^,^^30^ with empirical AUC being the reader performance metric. The study compared the relative performance of single spin-echo magnetic resonance imaging (SE MRI) to cinematic presentation of MRI (CINE MRI) for the detection of thoracic aortic dissection. There were $n_{0}=69$ patients without a dissection and $n_{1}=45$ patients with an aortic dissection imaged with both SE MRI and CINE MRI; cases were evaluated by five readers using a five-point ordinal confidence-of-disease scale. Similarly, each RMH simulated sample emulated five readers, each evaluating the same 69 nondiseased and 45 diseased cases.

We apply the OR-to-RMH algorithm to the set of parameter estimates (“original” values) obtained from an OR analysis of the data set to obtain corresponding RMH parameters values, simulate 10,000 MRMC samples based on the RMH values and analyze each simulated sample using an OR analysis, using the unbiased error covariance method, with the outcome being the empirical AUC. We set b_method = unspecified for the OR-to-RMH algorithm.

**Fig. 4:**
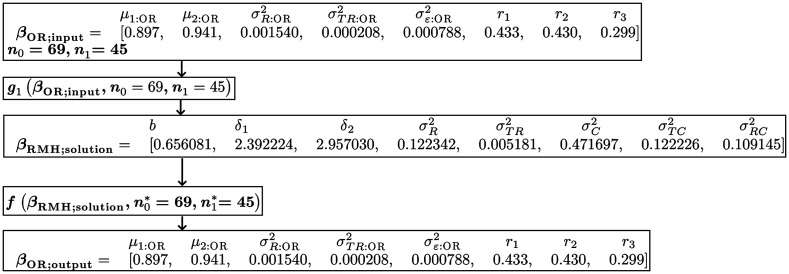
Flowchart, analogous to Fig. 2, illustrating the use of the OR-to-RMH and RMH-to-OR algorithms to simulate MRMC data that emulate the VanDyke^28^ data.

Figure 4 shows the computation of the RMH simulation model and the “true values,” which we define as the OR parameter values that describe the true distribution of the empirical AUCs computed from the simulated samples; i.e., the true values are the same as the outputted OR parameter values, given by $\beta_{\mathrm{OR};\text{output}}$. We see that for this data set the outputted values are the same as the inputted values, and hence the original OR estimates exactly describe the true distribution of the simulated empirical AUC estimates. The R code and output for the OR-to-RMH and RMH-to-OR functions used to produce the results in Fig. 4 are provided in Appendix C.1.

#### 4.2.2 Simulation study results

Table 1 presents the simulation study results. “Unbiased estimates” are the empirical estimates (the means across the simulated sample estimates) for the first eight parameters ($\mu_{1;\mathrm{OR}}$, $\mu_{2;\mathrm{OR}}$, $\sigma_{R:\mathrm{OR}}^{2},\ldots ,{\mathrm{Cov}}_{3}$), where OR estimates for each sample were computed using the OR method with the unbiased covariance estimation method discussed in Sec. 2.2. Because the sample estimates for the sample-level correlations $r_{1},r_{2}$, and $r_{3}$ are not unbiased, instead of reporting the empirical estimates we report the quotients resulting from dividing the corresponding empirical covariance estimates by the empirical error variance estimate. For example, the estimate of 0.434 for $r_{1}$ is computed by dividing the ${\mathrm{Cov}}_{1}$ estimate (0.000343) by the $\sigma_{\varepsilon :OR}^{2}$ estimate (0.000791). Because the resulting estimates are not the means of the sample-level correlations, empirical bias estimates and 95% confidence intervals for the correlations are not included.

**Table 1:** Simulation study estimates of OR parameters.

	OR parameters										
	$\mu_{1;OR}$	$\mu_{2;OR}$	$\sigma_{R:\mathrm{OR}}^{2}$	$\sigma_{TR:\mathrm{OR}}^{2}$	$\sigma_{\varepsilon :\mathrm{OR}}^{2}$	${\mathrm{Cov}}_{1}$	${\mathrm{Cov}}_{2}$	${\mathrm{Cov}}_{3}$	$r_{1}$	$r_{2}$	$r_{3}$
True values	0.897	0.941	0.001540	0.000208	0.000788	0.000341	0.000339	0.000236	0.433	0.430	0.299
Unbiased-method estimates	0.897	0.941	0.001537	0.000211	0.000791	0.000343	0.000341	0.000238	0.434	0.432	0.301
(Est - true)/true	−0.03%	−0.04%	−0.18%	1.38%	0.40%	0.59%	0.78%	0.93%	0.19%	0.38%	0.53%
Within 95% CI?	Yes	Yes	Yes	Yes	Yes	Yes	Yes	Yes	—	—	—
DeLong estimates	—	—	0.001537	0.000201	0.000802	0.000344	0.000343	0.000238	0.429	0.427	0.297
(Est - true)/true	—	—	−0.21%	−3.15%	1.80%	0.91%	1.12%	1.20%	−0.87%	−0.67%	−0.59%
Within 95% CI?	—	—	Yes	No	No	No	No	Yes	—	—	—

Notes: There were 10,000 simulated samples based on the Fig. 4 RMH model with 5 readers and $n_{0}=69$, $n_{1}=45$. “True values” are the $\beta_{\mathrm{OR};\text{output}}$ values from Fig. 4 and the corresponding error variance and covariances. For the first eight parameters, “unbiased-method estimates” and ” DeLong estimates” are the empirical estimates (i.e., means across the 10,000 samples) corresponding to using unbiased and DeLong error covariance estimation methods with the OR method. The correlation estimates are the quotients from dividing the corresponding covariance empirical covariance estimates by the empirical error variance estimate. “Within 95% CI?” is “yes” if the 95% confidence interval includes the true value and is “no” otherwise. For the DeLong estimates, results for $\mu_{1:\mathrm{OR}}$ and $\mu_{2:\mathrm{OR}}$ are omitted since they are exactly the same as for the unbiased estimates.

“(Est - true)/true” is defined as (estimate – true value)/(true value); it describes the deviation of the estimate from the true value and is expressed as a percentage of the true value. For the first eight parameters (i.e., not the correlations), these values can also be interpreted as the empirical estimates of statistical bias expressed as a percentage of the true value. “Within 95% CI?” is “yes” if the empirical 95% confidence interval (not shown) includes the true value, and otherwise is “no.”

We see that the unbiased estimates for the first eight parameters differ by <1.38% from the true values and that the correlation estimates differ by <0.53%. Moreover, all of the 95% empirical confidence intervals include the true value. Thus, the unbiased estimates agree with the true parameter values and hence provide validation for the OR-to-RMH algorithm.

**Fig. 5:**
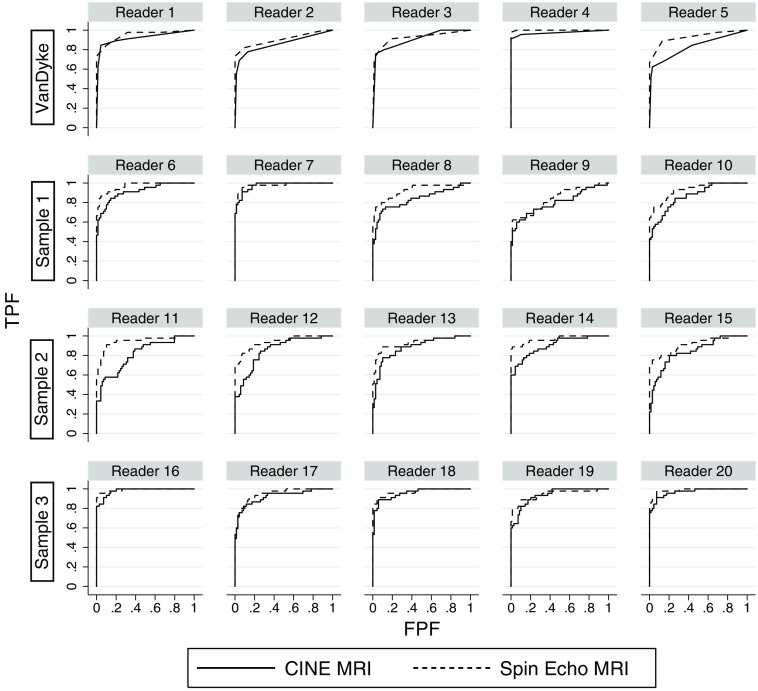
Comparison of empirical ROC curves computed from VanDyke data and three MRMC data samples that emulate the VanDyke data, generated from the RMH model in Fig. 4. TPF, true positive fraction (or sensitivity); FPF, false positive fraction (or 1 – specificity).

Plots of the empirical ROC curve for the VanDyke original data and for the first three simulated MRMC samples, based on the RMH model given in Fig. 4, are displayed in Fig. 5. Like the VanDyke study, each simulated sample has five independent readers reading the same set of 69 nondiseased and 45 diseased cases. Although the plots look somewhat different because the VanDyke plots are based on at most five distinct ratings, whereas the simulated-data plots are based on a continuous rating scale, in general the simulated-data ROC curves show a definite resemblance to the VanDyke ROC curves, although this is only our subjective assessment.

### 4.3 Other Remarks and Examples

#### 4.3.1 DeLong error covariance estimation

For comparison, we also include in Table 1 results using the DeLong et al.^12^ (DeLong) error covariance estimation method. Results for $\mu_{1:\mathrm{OR}}$ and $\mu_{2:\mathrm{OR}}$ are omitted since they depend only on the AUC estimation method and hence remain the same. We see from the confidence intervals that DeLong estimates for $\sigma_{\varepsilon }^{2}$, ${\mathrm{Cov}}_{1}$, and ${\mathrm{Cov}}_{2}$ are positively biased and the $\sigma_{TR}^{2}$ estimate is negatively biased. Similar results were obtained by Hillis.^6^ Although the DeLong method is biased, the estimates are relatively close to the true values, suggesting that results using the DeLong or another resampling error-covariance method, such as the jackknife or bootstrap, will typically be similar to those obtained using the unbiased method. This point is illustrated by the example in the next section.

#### 4.3.2 Example of computing power

Suppose our goal is to estimate the power for detecting a difference in test AUCs for a study such as the VanDyke study, assuming that the reader-averaged empirical AUC estimates (0.897 and 0.941) are the true population values. This can be done by simulating similar data (as we did for Table 1) and then estimating power by the proportion of samples where the null hypothesis is rejected. The power estimates from doing this, based on the simulated samples used for Table 1, are 0.106 for the unbiased method and 0.107 using the DeLong method, illustrating how the choice of error covariance method makes almost no difference in our power estimates.

#### 4.3.3 Ordinal rating scale

A limitation of the OR-to-RMH algorithm is that it applies only to continuous simulated ratings. For example, in Sec. 4, the simulation data emulated a continuous rating for which the empirical AUC distribution could be described by the original OR parameter values, but the VanDyke data set that yielded the original OR estimates consisted of ratings on a five-point ordinal scale. Although ordinal data can be simulated based on the RMH model by binning the simulated continuous data, the mapping from the RMH model to the corresponding OR parameters when the data are binned has not yet been developed, and hence neither has the corresponding OR-to-RM algorithm been developed.

We conducted a simulation study to investigate how close the original OR parameter values might describe the distribution of the empirical AUC for ordinal ratings resulting from binning the continuous ratings generated by the RMH model given in Fig. 4. The simulation study was performed similar to Table 1 study, except that five-category ordinal ratings were created by binning simulated continuous ratings. The binning thresholds corresponded to the empirical cumulative probabilities for ratings 1,…,5 for the VanDyke nondiseased cases, pooled across readers.

Results are presented in Table 2. As expected, the two AUC ($\mu_{1:\mathrm{OR}},\mu_{2:\mathrm{OR}}$) estimates are less than for the continuous values, but only by a maximum of 1.44%. We also see that the correlations are similar to those for the continuous ratings (maximum deviation is −4.63%), with the relative values of the three even more similar: $r_{1}\approx r_{2}$, as was the case for the continuous ratings, and $r_{3}$ is 0.12 lower than the other two, compared to being 0.13 lower for the continuous ratings. The maximum change in the error variance and covariance estimates was 8.07% and there were 6.7% and −7.9% changes in $\sigma_{R:\mathrm{OR}}^{2}$ and $\sigma_{TR:\mathrm{OR}}^{2}$, respectively, which are in the same “ballpark” as for the continuous ratings.

**Table 2:** Simulation results when continuous ratings are binned into a five-point ordinal scale.

	OR parameters										
	$\mu_{1:\mathrm{OR}}$	$\mu_{2:\mathrm{OR}}$	$\sigma_{R:\mathrm{OR}}^{2}$	$\sigma_{TR:\mathrm{OR}}^{2}$	$\sigma_{\varepsilon :\mathrm{OR}}^{2}$	${\mathrm{Cov}}_{1}$	${\mathrm{Cov}}_{2}$	${\mathrm{Cov}}_{3}$	$r_{1}$	$r_{2}$	$r_{3}$
True values (same as in Table 1)	0.897	0.941	0.001540	0.000208	0.000788	0.000341	0.000339	0.000236	0.433	0.430	0.299
Unbiased-method estimates from binned ratings	0.884	0.930	0.001643	0.000192	0.000852	0.000352	0.000352	0.000250	0.413	0.414	0.293
(Est - true)/true	−1.44%	−1.18%	6.67%	−7.90%	8.07%	3.07%	3.98%	5.96%	−4.63%	−3.78%	−1.95%

Notes: See notes for Table 1. OR parameter estimates are based on five-category ordinal ratings resulting from binning the continuous simulated ratings using the thresholds −0.2085494, 1.0270435, 1.7437654, and 2.3781446; these thresholds correspond to the empirical cumulative probabilities 0.4174, 0.8478, 0.9594, and 0.9913 for ratings 1 to 5 that were computed from the VanDyke data.

We conclude that compared with the continuous data, the empirical AUC distribution for the binned data has a similar correlation structure, similar AUC estimates and somewhat similar values for the error variance, error covariances, $\sigma_{R:\mathrm{OR}}^{2}$ and $\sigma_{TR:\mathrm{OR}}^{2}$. Thus, this example shows that the simulated ordinal data approximately emulate the VanDyke data set. Moreover, one could adjust the RMH parameters to result in a closer emulation using an iterative approach, where each iteration consists of adjustment of original OR values based on results from the previous-iteration simulation study, computation of corresponding RMH values, and a corresponding simulation study. For example, a first iteration might begin by upward adjustment of the $\mu_{1:\mathrm{OR}}$ and $\mu_{2:\mathrm{OR}}$ values.

#### 4.3.4 Changing the numbers of readers and cases

In our examples, thus far we have set the numbers of readers, diseased cases, and nondiseased cases to be the same as those of the VanDyke data set. However, often a researcher will want to investigate the performance of a reader-performance metric for a range of these numbers.

##### Readers

For a given set of RMH parameter values, changing the number of readers has no effect on the corresponding OR parameters $\mu_{1:\mathrm{OR}}$, $\mu_{2:\mathrm{OR}}$, $\sigma_{R:\mathrm{OR}}^{2}$, $\sigma_{TR:\mathrm{OR}}^{2}$, $\sigma_{\varepsilon :\mathrm{OR}}^{2}$, ${\mathrm{Cov}}_{1}$, ${\mathrm{Cov}}_{2}$, ${\mathrm{Cov}}_{3}$, $r_{1}$, $r_{2}$, and $r_{3}$, as shown by the omission of the reader number in the RMH-to-OR algorithm formulas in Table 6 in Appendix A.

##### Cases

For a given set of RMH parameter values, changing the number of cases has no effect on $\mu_{1:\mathrm{OR}}$, $\mu_{2:\mathrm{OR}}$, $\sigma_{R:\mathrm{OR}}^{2}$, or $\sigma_{TR:\mathrm{OR}}^{2}$, as shown by the omission of the case sample sizes in the corresponding formulas in Table 6. In contrast, $\sigma_{\varepsilon :\mathrm{OR}}^{2},{\mathrm{Cov}}_{1},{\mathrm{Cov}}_{2}$, and ${\mathrm{Cov}}_{3}$ will be affected. Although the correlations are also affected, changes in the correlations will typically be small [Ref. 6, p 2078].

For example, Table 3(b) shows when the case sizes are doubled ($n_{0}=138$, $n_{1}=90$) that $\sigma_{\varepsilon :\mathrm{OR}}^{2}$ is reduced by 50%, the correlations are virtually unchanged (maximum of 0.6%), and there is no change in $\sigma_{R:\mathrm{OR}}^{2}$, $\sigma_{TR:\mathrm{OR}}^{2}$, $q_{1}$ or $q_{2}$. Table 3c shows when the case sample sizes are switched ($n_{0}=45$, $n_{1}=69$) that $\sigma_{\varepsilon :\mathrm{OR}}^{2}$ is reduced by 19% and there is a small increase in the correlations (maximum increase of 2.3%), with all other values remaining unchanged. These results are computed using the RMH-to-OR formulas in Table 6, thus eliminating the need for simulations.

**Table 3:** Effect of different case sizes and RMH $\delta_{1}$ and $\delta_{2}$ values on OR parameters.

Change from OR true values	$q_{1}$	$q_{2}$	Pr1	$\mu_{1:\mathrm{OR}}$	$\mu_{2:\mathrm{OR}}$	$\sigma_{R:\mathrm{OR}}^{2}$	$\sigma_{TR:\mathrm{OR}}^{2}$	$\sigma_{\varepsilon :\mathrm{OR}}^{2}$	$r_{1}$	$r_{2}$	$r_{3}$
(a) No change (true values from Fig. 4)	4.56	5.64	0.004	0.897	0.941	0.00154	0.000208	0.000788	0.433	0.430	0.299
(b) Case sizes doubled $\left(n_{0}=138,n_{1}=90\right)$	4.56	5.64	0.004	0.897	0.941	0.00154	0.000208	0.000391	0.435	0.432	0.301
	—	—	—	—	—	(0.00%)	(0.00%)	(−50%)	(0.5%)	(0.5%)	(0.6%)
(c) Case sizes switched $\left(n_{0}=45,n_{1}=69\right)$	4.56	5.64	0.004	0.897	0.941	0.00154	0.000208	0.000634	0.441	0.438	0.306
	—	—	—	—	—	(0.00%)	(0.00%)	(−19%)	(2.0%)	(1.8%)	(2.3%)
(d) Null model 1 $\left(\delta_{1}=\delta_{2}=2.6452\right)$	5.05	5.05	0.001	0.919	0.919	0.00164	0.000074	0.000789	0.462	0.426	0.319
	—	—	—	—	—	(6.8%)	(−64%)	(0.13%)	(6.7%)	(−0.9%)	(6.6%)
(e) Null model 2 $\left(\delta_{1}=\delta_{2}=1.2759\right)$	2.43	2.43	0.326	0.75	0.75	0.00701	0.000302	0.002346	0.522	0.515	0.401
	—	—	—	—	—	(355%)	(45%)	(198%)	(21%)	(20%)	(34.1%)

Notes: Part (a) shows the set of OR parameter “true values” ($\beta_{\mathrm{OR};\text{solution}}$) from Fig. 4 that correspond to simulations using the RMH model parameters ($\beta_{\mathrm{RMH};\text{solution}}$) in Fig. 4 when $n_{0}=69$, and $n_{1}=45$. In addition, the median mean-to-sigma ratios $q_{1}$ and $q_{2}$ corresponding to the test 1 and 2 latent RMH rating distributions are included, as well as Pr1, defined as the probability that a reader’s true ROC curve is noticeably improper for test 1. Parts (b)–(e) show the corresponding values when the indicated changes are made to the case sizes for the simulated samples or to the RMH model values. The OR values are computed by applying the RMH-to-OR algorithm to the RMH model from Fig. 4 with the changes in the left column incorporated. Values in parentheses are the percentage change in the OR parameters from the original values. See Appendix C.2 for the R code that produced these results.

#### 4.3.5 Null and power simulations

The example in Sec. 4.3.2 showed how power could be easily computed for simulated data that emulate a particular study, assuming the effect size ($\mu_{1:\mathrm{OR}}-\mu_{2:\mathrm{OR}}$) is equal to the observed effect size. Other effect sizes can be investigated by adjusting $\delta_{1}$ and $\delta_{2}$ in the RMH parameter set accordingly, using the relationship (from Table 6):

$$\mu_{i:\mathrm{OR}}=\Phi (\frac{\delta_{i}}{\sqrt{1+b^{-2}+2(\sigma_{R}^{2}+\sigma_{TR}^{2})}}),$$

which implies

$$\delta_{i}=\Phi^{-1}(\mu_{i:\mathrm{OR}})\sqrt{1+b^{-2}+2(\sigma_{R}^{2}+\sigma_{TR}^{2})},$$ (22)

where Φ is the cumulative standard normal distribution function.

In addition, often the researcher wants to empirically compute the type I error for testing $H_{0}:\mu_{1:\mathrm{OR}}=\mu_{2:\mathrm{OR}}$ versus $H_{0}:\mu_{1:\mathrm{OR}}\neq \mu_{2:\mathrm{OR}}$. This can be done by creating a null RMH model by setting $\delta_{1}=\delta_{2}$, with the empirical type I error rate given by the proportion of simulated samples where $H_{0}$ is rejected. For example, in Table 3(d) we alter the RMH model given in Fig. 4 by setting $\delta_{1}=\delta_{2}=\delta$, with the value of δ determined such that the corresponding $\mu_{i:\mathrm{OR}}$ values are both equal to $\mu_{\mathrm{OR}}=0.919$, the mean of the two original OR AUC values, 0.897 and 0.941, in Fig. 4. It follows from Eq. (22), with $\mu_{\mathrm{OR}}=(0.897+0.941)/2$, that δ=2.6452, using the values for $b,\sigma_{R}^{2}$, and $\sigma_{TR}^{2}$, given in Fig. 4.

In Table 3(e), we similarly determine for a null RMH model the value of δ that correspond to $\mu_{\mathrm{OR}}=0.75$. In both Table 3(d) and 3(e), we see that all of the original OR parameter values are changed, as well as the mean-to-sigma ratios, with Table 3(e) showing much more change. For this reason, we suggest that if the researcher wants to simulate data with error correlations and reader and reader-by-test variance components similar to those from an OR analysis of a real-data study, but with much different AUC values, the OR-to-RMH algorithm with b_option = mean_to_sigma should be used to determine the corresponding $\beta_{\mathrm{RM}}$ vector, as discussed in the next section.

The R code and output for the OR-to-RMH and RMH-to-OR functions used to produce the results in Table 3 are included in Appendix C.2.

#### 4.3.6 Mean-to-sigma ratios and the specified and mean_to_sigma b_options

From Table 3, parts (a)-(c), we see that the mean-to-sigma ratios are $q_{1}=4.56$ and $q_{2}=5.64$ for the Fig. 4 RMH model latent distributions, as well as for the models when the case sizes are changed. However, in parts (d) and (e), we see that when the values for the RMH parameters $\delta_{1}$ and $\delta_{2}$ are changed, the mean-to-sigma ratios also change.

In Table 3, Pr1 is the probability that a reader’s true ROC curve is noticeably improper for test 1. (See Appendix B.1 for how to compute Pr1.) We see that this probability is relatively small (≤0.004) for the first four models and thus is not of concern. In contrast, Pr1 = 0.326 for null model 2, and thus we recommend not using this model for a simulation study. (Note: although Pr2, the analogous probability for test 2, is not included in Table 3, conclusions based on it were the same.)

In Table 4, we see for the specified and mean_to_sigma b_methods that the OR parameters corresponding to the resulting RMH models are equal to all of the original OR values except for the error variance and covariances (not shown).

**Table 4:** Comparison of RMH parameter values and corresponding true OR values resulting from using the three different b_methods. The RMH parameter values ($\beta_{\mathrm{RMH};\text{solution}}$) are obtained by applying the OR-to-RMH algorithm to the “original” OR parameter values ($\beta_{\mathrm{OR};\text{input}}$) in Fig. 4. The true OR values ($\beta_{\mathrm{OR};\text{output}}$) result from applying the RMH-to-OR algorithm to the RMH parameter values. See Appendix C.4 for the corresponding R code and the complete sets of RMH and OR values.

b_method	$n_{0}$	$n_{1}$	RMH parameter values				True OR values							
			b	$q_{1}$	$q_{2}$	Pr1	$\mu_{1:\mathrm{OR}}$	$\mu_{2:\mathrm{OR}}$	$\sigma_{R:\mathrm{OR}}^{2}$	$\sigma_{TR:\mathrm{OR}}^{2}$	$\sigma_{\varepsilon :\mathrm{OR}}^{2}$	$r_{1}$	$r_{2}$	$r_{3}$
unspecified	69	45	0.65608	4.56	5.64	0.004	0.897	0.941	0.00154	0.000208	0.000788	0.433	0.43	0.299
mean_to_sigma ^a^	69	45	0.69297	5.20	6.43	0.002	0.897	0.941	0.00154	0.000208	0.000766	0.433	0.43	0.299
Specified ^b^	69	45	1	∞	∞	0.000	0.897	0.941	0.00154	0.000208	0.000658	0.433	0.43	0.299

a Used with *mean_sig_input* = 5.2.

b Used with *b_input* = 1.

The R code for generating Table 4 is included in Appendix C.3.

#### 4.3.7 Troubleshooting

Table 5 provides examples where the OR-to-RMH algorithm fails to produce a solution. In each example, the OR-to-RMH algorithm is applied to the original parameter estimate values from the VanDyke study, given in Fig. 4, but with one value altered to result in the algorithm not working. For example, in part (a) $\sigma_{R:\mathrm{OR}}^{2}$ is changed from 0.00154 (original value) to 0.154 and the algorithm fails. Using Table 11 in Appendix A, we can identify which input value is causing the problem by checking for the first parameter in the sequence $x_{1},x_{2},x_{3},x_{4},b,x_{5},x_{6},x_{7}$ that is missing (NA), where $x_{1},\ldots ,x_{7}$ are the alternative RM parameters discussed in Appendix A. Noting that the first parameter with a missing value is $x_{3}$, the rules in Table 11 suggest reducing the value of $\sigma_{R:\mathrm{OR}}^{2}.$ Similarly, in part (b), $\sigma_{TR:\mathrm{OR}}^{2}$ is increased and $x_{4}$ is the first parameter with a missing value; here, Table 11 suggests reducing the value of $\sigma_{TR:\mathrm{OR}}^{2}.$ In part (c), $\sigma_{\varepsilon :\mathrm{OR}}^{2}$ is increased and b is the first parameter with a missing value; here, Table 11 suggests either changing (reducing or increasing) the value of $\sigma_{\varepsilon ;\mathrm{OR}}^{2}$ or using b_method = specified or b_method = mean_to_sigma.

The R code for generating the results in Table 5 is provided in Appendix C.4. The values for the $x_{1},\ldots ,x_{7}$ parameters are by default not printed unless the option all = T is included in the print function, as illustrated in Appendix C.4. Also note in Appendix C.4 that the *OR_to_RM* function suggests the remedy, based on Table 11, when the algorithm fails to produce a solution.

**Table 5:** Troubleshooting examples. For each example, one of the original parameter estimate values from the VanDyke study, as given by $\beta_{\mathrm{OR};\mathrm{orig}}$ in Fig. 4, is replaced by a value that causes the OR-to-RMH algorithm to fail. These examples show how the value responsible for the algorithm failure can be identified from the alternative parameters $x_{1},\ldots ,x_{7}$ and b values using the rules given Table 11. All examples use b_method = unspecified. See Appendix C.4 for the R code that produced these results. Note that to print the $x_{1},\ldots ,x_{7}$ variables the option all = T must be included in the print function (see Appendix C.4 for examples).

(a) Original value: $\sigma_{R:\mathrm{OR}}^{2}=0.00154$. Altered value: $\sigma_{R:\mathrm{OR}}^{2}=0.154$. Output from applying OR-to-RMH algorithm to altered $\beta_{\mathrm{OR}:\mathrm{orig}}$ vector is shown below. Noting that $x_{3}$ is the first parameter in the sequence $x_{1},x_{2},x_{3},x_{4},b,x_{5},x_{6},x_{7}$ that is missing (NA), the rules in Table 11 suggest reducing the value of $\sigma_{R:\mathrm{OR}}^{2}$.
n0 n1 mu1 mu2 var_R var_TR var_C var_TC var_RC var_error
69 45 NA NA NA NA NA NA NA NA
b_method mean_sig1 mean_sig2 mean_sig1_025 mean_sig2_025
unspecified NA NA NA NA
x1 x2 x3 x4 b x5 x6 x7
1.264641 1.563224 NA NA NA NA NA NA
(b) Original value: $\sigma_{TR:\mathrm{OR}}^{2}=0.00028$. Altered value: $\sigma_{TR:\mathrm{OR}}^{2}=0.28$. Output from applying OR-to-RMH algorithm to altered $\beta_{\mathrm{OR}:\mathrm{orig}}$ vector is shown below. Noting that $x_{4}$ is the first parameter in the sequence $x_{1},x_{2},x_{3},x_{4},b,x_{5},x_{6},x_{7}$ that is missing (NA), the rules in Table 11 suggest reducing the value of $\sigma_{TR:\mathrm{OR}}^{2}$.
n0 n1 mu1 mu2 var_R var_TR var_C var_TC var_RC var_error
69 45 NA NA NA NA NA NA NA NA
b_method mean_sig1 mean_sig2 mean_sig1_025 mean_sig2_025
unspecified NA NA NA NA
x1 x2 x3 x4 b x5 x6 x7
1.264641 1.563224 0.06838082 NA NA NA NA NA
(c) Original value: $\sigma_{\varepsilon :\mathrm{OR}}^{2}=0.000788$. Altered value: $\sigma_{\varepsilon :\mathrm{OR}}^{2}=0.00788$. Output from applying OR-to-RMH algorithm to altered $\beta_{\mathrm{OR}:\mathrm{orig}}$ vector is shown below. Noting that b is the first parameter in the sequence $x_{1},x_{2},x_{3},x_{4},b,x_{5},x_{6},x_{7}$ that is missing (NA), the rules in Table 11 suggest either changing (reducing or increasing) the value of $\sigma_{\varepsilon :\mathrm{OR}}^{2}$, or using b_method = specified or b_method = mean_to_sigma
n0 n1 mu1 mu2 var_R var_TR var_C var_TC var_RC var_error
69 45 NA NA NA NA NA NA NA NA
b_method mean_sig1 mean_sig2 mean_sig1_025 mean_sig2_025
unspecified NA NA NA NA

#### 4.3.8 Using the algorithm with Gallas parameter estimates

For a real-data MRMC study analyzed by the Gallas method,^15^^,^^31^ a method has been developed to convert the U-statistic parameters of empirical AUC and variance estimates to RM model parameters.^32^ Alternatively, it has been shown by Hillis^14^ that the Gallas MRMC method produces the same empirical AUC single test and difference-of-two-tests variance estimates as the OR method, if the constraints given by Eq. (12) are not imposed on the OR estimates. As a result, OR parameter estimates can be computed from the Gallas parameter estimates using formulas provided in Hillis.^14^ Hence, RMH model parameters that correspond to real data studies can be derived using the OR-to-RMH algorithm applied to the transformed Gallas parameter estimates.

## 5 Discussion

A previous problem with the original RM model and later generalized versions of it was that the RM model parameters were expressed only in terms of the latent binormal rating distributions, as opposed to the more familiar reader performance measure distributions. Thus, it has been difficult to set RM model parameters such that the simulated data were similar to MRMC data encountered in practice. Assuming the constrained unequal-variance RM model,^3^ which we have referred to as the RMH model in this paper, Hillis^6^ recently remedied this problem by deriving formulas for computing the OR parameter values that describe the distribution of empirical AUC outcomes computed from RMH simulated data. However, that paper did not provide a reverse OR-to-RMH mapping. This paper overcomes that limitation by deriving a numerical OR-to-RMH algorithm that computes RMH parameter values from a specified set of OR parameter values and by providing an R function to implement the algorithm. The OR-to-RMH algorithm and its corresponding R function make it easy to calibrate the RMH model to produce simulated data that emulate specific real data sets with respect to the distribution of the empirical AUC estimates.

The original RM model paper^2^ presented several simulation structures that were supposed to represent ROC analyses of representative real data sets, which was useful because then researchers could assess the performance of MRMC analysis methods using a commonly accepted set of RM simulation structures. However, there was a mistake in some of the computations of the RM parameters and the model was limited to equal-variance binormal ROC curves, which are not common.^6^

The present approach has several limitations that we hope to remedy in future research. It is limited to generating continuous rating data that emulate a set of inputted OR parameter values describing the distribution of the empirical AUC estimates. Although the simulated continuous rating data can be binned, the distribution of the empirical AUC estimates for the binned data will not as closely emulate the inputted OR parameter values. We suggested a method to adjust the parameter values to better fit ordinal discrete ratings through an iterative simulation approach, but this process is time consuming and we hope to develop RMH-to-OR and OR-to-RMH algorithms, similar to the ones in this paper, that are primarily designed for simulation of rating data with a few ordinal values (e.g., 1, 2, 3, 4, or 5).

The present approach is also limited to the empirical AUC as the reader performance measure. We hope to develop an approach that allows for a semiparametric outcome, such as the binormal AUC.

Finally, our algorithm is based on the RMH model,^3^ which assumes that the latent distributions are the same for both tests. Thus, another area for future research is to relax this assumption and develop algorithms for a more general RM model, such as the unconstrained unequal variance model,^6^ the generalized RM model,^5^ or some other generalization of the original RM model.

## 6 Conclusions

The main contributions of this paper are the OR-to-RMH algorithm and the corresponding R software OR_to_RMH function; these contributions make it easy to calibrate RMH model parameters to match real-data OR parameter estimates, thus making it easy to simulate rating data that emulate real data sets for testing MRMC analysis methods or for performing power analysis. These contributions will allow researchers to develop sets of RMH simulation structures that are representative of a wide spectrum of MRMC studies, which can then be used to validate MRMC analysis methods. We expect these new RMH simulation structures will replace the original RM model structures, which were not linked to specific real-world data sets and were limited to equal-variance ROC curves, making the representativeness of the structures difficult to evaluate.

## 7 Appendix A: Algorithm Details for Mapping OR Model Parameters to RMH model Parameters

In this section, we derive the mapping from OR model parameters to RMH model parameters. For the mapping, we assume the RMH model because it has the same number of parameters as the OR model. The mapping from a more general RM model, which includes the RMH model as a special case, to the OR model was derived by Hillis.^6^ Modifying this more RM general model by constraining the error variance and variance components involving diseased cases to be equal to those involving nondiseased cases multiplied by $b^{2},b>0$, results in the RMH model. Table 6 presents the resulting analytical RMH-to-OR mapping.

To facilitate the derivation of the reverse (OR-to-RM) mapping, an alternative parameterization for the RMH model is presented in Table 7. Table 7(a) expresses the alternative RMH parameters in terms of the RMH parameters, Table 7(b) presents the constraints on these parameters, and Table 7(c) expresses the RMH model parameters in terms of the alternative RMH parameters. Table 8 expresses the OR parameters in terms of the alternative RMH parameters and Table 9 expresses the alternative RMH parameters in terms of the OR parameters.

**Table 6:** RMH-to-OR mapping: OR parameters expressed in terms of the RMH model parameters for the empirical AUC.

$\mu_{i:\mathrm{OR}}=\Phi (\frac{\delta_{i}}{\sqrt{V}}),\;i=1,2$
$\sigma_{R:\mathrm{OR}}^{2}=F_{\mathrm{BVN}}(\frac{\delta_{1}}{\sqrt{V}},\frac{\delta_{2}}{\sqrt{V}};\frac{2\sigma_{R}^{2}}{V})-[\Phi (\frac{\delta_{1}}{\sqrt{V}})\Phi (\frac{\delta_{2}}{\sqrt{V}})]$
$\sigma_{TR:\mathrm{OR}}^{2}=.5\overset{2}{\underset{i=1}{\sum }}\{F_{\mathrm{BVN}}(\frac{\delta_{i}}{\sqrt{V}},\frac{\delta_{i}}{\sqrt{V}};\frac{2(\sigma_{R}^{2}+\sigma_{TR}^{2})}{V})-{\left[\Phi (\frac{\delta_{i}}{\sqrt{V}})\right]}^{2}\}-\sigma_{R:\mathrm{OR}}^{2}$
${\mathrm{Cov}}_{1}=\overset{4}{\underset{m=1}{\sum }}c_{m}F_{\mathrm{BVN}}(\frac{\delta_{1}}{\sqrt{V}},\frac{\delta_{2}}{\sqrt{V}};\frac{\rho_{m}(1+b^{-2})+2\sigma_{R}^{2}}{V})$
where $\rho_{1}=\frac{\sigma_{RC(-)}^{2}+\sigma_{C(-)}^{2}+\sigma_{RC(+)}^{2}+\sigma_{C(+)}^{2}}{1+b^{-2}},\;\rho_{2}=\frac{\sigma_{RC(-)}^{2}+\sigma_{C(-)}^{2}}{1+b^{-2}},\;\rho_{3}=\frac{\sigma_{RC(+)}^{2}+\sigma_{C(+)}^{2}}{1+b^{-2}},\;\rho_{4}=0$
${\mathrm{Cov}}_{2}=\frac{1}{2}\overset{2}{\underset{i=1}{\sum }}\overset{4}{\underset{m=1}{\sum }}c_{m}F_{\mathrm{BVN}}(\frac{\delta_{i}}{\sqrt{V}},\frac{\delta_{i}}{\sqrt{V}};\frac{\rho_{m}(1+b^{-2})}{V})$
$\rho_{1}=\frac{\sigma_{C(-)}^{2}+\sigma_{TC(-)}^{2}+\sigma_{C(+)}^{2}+\sigma_{TC(+)}^{2}}{1+b^{-2}},\;\rho_{2}=\frac{\sigma_{C(-)}^{2}+\sigma_{TC(-)}^{2}}{1+b^{-2}},\;\rho_{3}=\frac{\sigma_{C(+)}^{2}+\sigma_{TC(+)}^{2}}{1+b^{-2}},\;\rho_{4}=0$
${\mathrm{Cov}}_{3}=\overset{4}{\underset{m=1}{\sum }}c_{m}F_{\mathrm{BVN}}(\frac{\delta_{1}}{\sqrt{V}},\frac{\delta_{2}}{\sqrt{V}};\frac{\rho_{m}(1+b^{-2})}{V})$
where $\rho_{1}=\frac{\sigma_{C(-)}^{2}+\sigma_{C(+)}^{2}}{1+b^{-2}},\;\rho_{2}=\frac{\sigma_{C(-)}^{2}}{1+b^{-2}},\;\rho_{3}=\frac{\sigma_{C(+)}^{2}}{1+b^{-2}},\;\rho_{4}=0$
$\sigma_{\varepsilon :\mathrm{OR}}^{2}=\frac{1}{2}\overset{2}{\underset{i=1}{\sum }}\overset{4}{\underset{m=1}{\sum }}c_{m}F_{\mathrm{BVN}}(\frac{\delta_{i}}{\sqrt{V}},\frac{\delta_{i}}{\sqrt{V}};\frac{\rho_{m}(1+b^{-2})+2(\sigma_{R}^{2}+\sigma_{TR}^{2})}{V})$
where $\rho_{1}=1,\;\rho_{2}=\frac{\sigma_{TC(-)}^{2}+\sigma_{RC(-)}^{2}+\sigma_{C(-)}^{2}+\sigma_{\varepsilon (-)}^{2}}{1+b^{-2}},\;\rho_{3}=\frac{\sigma_{TC(+)}^{2}+\sigma_{RC(+)}^{2}+\sigma_{C(+)}^{2}+\sigma_{\varepsilon (+)}^{2}}{1+b^{-2}},\;\rho_{4}=0$

Notes: The numbers of nondiseased and diseased cases are denoted by $n_{0}$ and $n_{1};F_{\mathrm{BVN}}(.,.;\rho )$ denotes the standardized bivariate normal distribution function with correlation ρ; $\mu_{i:\mathrm{OR}}=\mu_{\mathrm{OR}}+\tau_{i:\mathrm{OR}}$; $\delta_{i}=\mu_{+}+\tau_{i+}$; $V=1+b^{-2}+2(\sigma_{R}^{2}+\sigma_{TR}^{2})$; $c_{1}=\frac{1}{n_{0}n_{1}}$; $c_{2}=\frac{n_{1}-1}{n_{0}n_{1}}$; $c_{3}=\frac{n_{0}-1}{n_{0}n_{1}}$; $c_{4}=\frac{1-n_{0}-n_{1}}{n_{0}n_{1}}$. This table is reprinted, adapted, and revised with permission from Hillis [Ref. 6, Table 3]; notation is the same except that $\left(\sigma_{\text{fixed}(-)}^{2}+\sigma_{\text{fixed}(+)}^{2}\right)$ has been replaced by $1+b^{-2},b>0$, which results in the RMH model.

**Table 7:** Alternative parameterization for RMH model parameterization.

(a) Alternative RMH parameters expressed in terms of RMH model parameters. V is defined by $V=1+b^{-2}+2(\sigma_{R}^{2}+\sigma_{TR}^{2})$. Note that $x_{6}=x_{5}+\sigma_{TC(-)}^{2}$, $x_{7}=x_{5}+\sigma_{RC(-)}^{2}$, and $1-x_{4}=[1+b^{-2}]/V$.		
Alternative RMH model parameters		RMH model parameters
b	=	b
$x_{1}$	=	$(\delta_{1})/\sqrt{V}$
$x_{2}$	=	$(\delta_{2})/\sqrt{V}$
$x_{3}$	=	$2\sigma_{R}^{2}/V$
$x_{4}$	=	$[2(\sigma_{R}^{2}+\sigma_{TR}^{2})]/V$
$x_{5}$	=	$\sigma_{C(-)}^{2}$
$x_{6}$	=	$\sigma_{TC(-)}^{2}+\sigma_{C(-)}^{2}$
$x_{7}$	=	$\sigma_{RC(-)}^{2}+\sigma_{C(-)}^{2}$
(b) Constraints on RMH alternative parameters. These follow from the equations in part (a), nonnegativity of the RMH variance components and constraints Eqs. (6) and (8). $0\leq x_{1};\;0\leq x_{2};\;0\leq x_{i}\leq 1,\;i=3,\ldots ,7;\;x_{4}\geq x_{3};\;x_{6}\geq x_{5};\;x_{7}\geq x_{5};\;x_{6}+x_{7}-x_{5}\leq 1$ (23)		
(c) RMH parameters expressed in terms of alternative RMH parameters. Note that in terms of the alternative parameterization, $V=(1+1/b^{2})/(1-x_{4})$.		
RMH parameter		Alternative RMH parameter
b	=	b
$\delta_{1}$	=	$x_{1}\sqrt{V}$
$\delta_{2}$	=	$x_{2}\sqrt{V}$
$\sigma_{R}^{2}$	=	$.5x_{3}V$
$\sigma_{TR}^{2}$	=	$.5V(x_{4}-x_{3})$
$\sigma_{C(-)}^{2}$	=	$x_{5}$
$\sigma_{TC(-)}^{2}$	=	$x_{6}-x_{5}$
$\sigma_{RC(-)}^{2}$	=	$x_{7}-x_{5}$
$\sigma_{\varepsilon (-)}^{2}$	=	$1-(x_{6}+x_{7}-x_{5})$

**Table 8:** RMH-to-OR mapping: OR parameters for the empirical AUC expressed in terms of the alternative parameterization of the RMH model given in Table 7.

$\mu_{i:\mathrm{OR}}=\Phi (x_{i}),i=1,2$
$\sigma_{R:\mathrm{OR}}^{2}=F_{\mathrm{BVN}}(x_{1},x_{2};x_{3})-[\Phi (x_{1})\Phi (x_{2})]$
$\sigma_{TR:\mathrm{OR}}^{2}=.5\overset{2}{\underset{i=1}{\sum }}\{F_{\mathrm{BVN}}(x_{i},x_{i};x_{4})-{\left[\Phi (x_{i})\right]}^{2}\}-\sigma_{R:\mathrm{OR}}^{2}$
$\sigma_{\varepsilon :\mathrm{OR}}^{2}=\frac{1}{2}\overset{2}{\underset{i=1}{\sum }}\overset{4}{\underset{m=1}{\sum }}c_{m}F_{\mathrm{BVN}}(x_{i},x_{i};\rho_{m}(1-x_{4})+x_{4})$
where $\rho_{1}=1,\;\rho_{2}=\frac{1}{1+b^{-2}},\;\rho_{3}=\frac{1}{1+b^{2}},\;\rho_{4}=0$
${\mathrm{Cov}}_{1}=\sum_{m=1}^{4}c_{m}F_{\mathrm{BVN}}(x_{1},x_{2};\rho_{m}(1-x_{4})+x_{3})$
where $\rho_{1}=x_{7},\;\rho_{2}=\frac{x_{7}}{1+b^{-2}},\;\rho_{3}=\frac{x_{7}}{\left(1+b^{2}\right)},\;\rho_{4}=0$
${\mathrm{Cov}}_{2}=.\frac{1}{2}\overset{2}{\underset{i=1}{\sum }}\overset{4}{\underset{m=1}{\sum }}c_{m}F_{\mathrm{BVN}}(x_{i},x_{i};\rho_{m}(1-x_{4}))$
where $\rho_{1}=x_{6},\;\rho_{2}=\frac{x_{6}}{1+b^{-2}},\;\rho_{3}=\frac{x_{6}}{1+b^{2}},\;\rho_{4}=0$
${\mathrm{Cov}}_{3}=\overset{4}{\underset{m=1}{\sum }}c_{m}F_{\mathrm{BVN}}(x_{1},x_{2};\rho_{m}(1-x_{4}))$
where $\rho_{1}=x_{5},\;\rho_{2}=\frac{x_{5}}{1+b^{-2}},\;\rho_{3}=\frac{x_{5}}{1+b^{2}},\;\rho_{4}=0$

Notes: This table results from replacing the RMH model parameters in Table 6 by the alternative RMH model parameters, as defined in Table 7(a). $F_{\mathrm{BVN}}(.,.;\rho )$ is the standardized bivariate normal distribution function with correlation ρ; $V=1+b^{-2}+2(\sigma_{R}^{2}+\sigma_{TR}^{2})$; $c_{1}=\frac{1}{n_{0}n_{1}}$; $c_{2}=\frac{n_{1}-1}{n_{0}n_{1}}$; $c_{3}=\frac{n_{0}-1}{n_{0}n_{1}}$; $c_{4}=\frac{1-n_{0}-n_{1}}{n_{0}n_{1}}$.

**Table 9:** Alternative RMH parameters expressed in terms of OR parameters.

$x_{1}=\Phi^{-1}(\mu_{1:\mathrm{OR}})$
$x_{2}=\Phi^{-1}(\mu_{2:\mathrm{OR}})$
$x_{3}=\{0\leq x_{3}\leq 1:\sigma_{R:\mathrm{OR}}^{2}-F_{\mathrm{BVN}}(x_{1},x_{2};x_{3})+\Phi (x_{1})\Phi (x_{2})=0\}$
$x_{4}=\{0\leq x_{4}\leq 1:\sigma_{TR:\mathrm{OR}}^{2}+\sigma_{R:\mathrm{OR}}^{2}-.5\overset{2}{\underset{i=1}{\sum }}\{F_{\mathrm{BVN}}(x_{i},x_{i};x_{4})-[\Phi (x_{i}){]}^{2}\}=0\}$
$b=\{b>0:\sigma_{\varepsilon :\mathrm{OR}}^{2}-\frac{1}{2}\overset{2}{\underset{i=1}{\sum }}\overset{4}{\underset{m=1}{\sum }}c_{m}F_{\mathrm{BVN}}(x_{i},x_{i};\rho_{m}(1-x_{4})+x_{4})=0\}$
where $\rho_{1}=1,\;\rho_{2}=\frac{1}{1+b^{-2}},\;\rho_{3}=\frac{1}{1+b^{2}},\;\rho_{4}=0$
$x_{5}=\{0\leq x_{5}\leq 1:{\mathrm{Cov}}_{3}-\overset{4}{\underset{m=1}{\sum }}c_{m}F_{\mathrm{BVN}}(x_{1},x_{2};\rho_{m}(1-x_{4}))=0\}$
where $\rho_{1}=x_{5},\;\rho_{2}=\frac{x_{5}}{1+b^{-2}},\;\rho_{3}=\frac{x_{5}}{1+b^{2}},\;\rho_{4}=0$
$x_{6}=\{0\leq x_{6}\leq 1:{\mathrm{Cov}}_{2}-\frac{1}{2}\overset{2}{\underset{i=1}{\sum }}\overset{4}{\underset{m=1}{\sum }}c_{m}F_{\mathrm{BVN}}(x_{i},x_{i};\rho_{m}(1-x_{4}))=0\}$
where $\rho_{1}=x_{6},\;\rho_{2}=\frac{x_{6}}{1+b^{-2}},\;\rho_{3}=\frac{x_{6}}{1+b^{2}},\;\rho_{4}=0$
$x_{7}=\{0\leq x_{7}\leq 1:{\mathrm{Cov}}_{1}-\overset{4}{\underset{m=1}{\sum }}c_{m}F_{\mathrm{BVN}}(x_{1},x_{2};\rho_{m}(1-x_{4})+x_{3})=0\}$
where $\rho_{1}=x_{7},\;\rho_{2}=\frac{x_{7}}{1+b^{-2}},\;\rho_{3}=\frac{x_{7}}{\left(1+b^{2}\right)},\;\rho_{4}=0$

Notes: These results follow from the Table 8 relationships and constraints Eq. (23) in Table 7. $F_{\mathrm{BVN}}(.,.;\rho )$ is the standardized bivariate normal distribution function with correlation ρ; $c_{1}=\frac{1}{n_{0}n_{1}}$; $c_{2}=\frac{n_{1}-1}{n_{0}n_{1}}$; $c_{3}=\frac{n_{0}-1}{n_{0}n_{1}}$; $c_{4}=\frac{1-n_{0}-n_{1}}{n_{0}n_{1}}$.

**Table 10:** OR-to-RMH algorithm for computing parameter values for the RMH model that correspond to specified OR parameter values.

**Step 1**. Solve for $x_{1}$ and $x_{2}:$
x1=Φ−1(θ^1) and x2=Φ−1(θ^2)
**Step 2**. Solve for $x_{3}$, using the values for $x_{1}$ and $x_{2}$ obtained in step 1:
$x_{3}=\{0\leq x_{3}<1:{\hat{\sigma }}_{R:\mathrm{OR}}^{2}-F_{\mathrm{BVN}}(x_{1},x_{2};x_{3})+\Phi (x_{1})\Phi (x_{2})=0\}$
From the relationship $F_{\mathrm{BVN}}(x,y;\rho_{1})<F_{\mathrm{BVN}}(x,y;\rho_{2})$ if $\rho_{1}<\rho_{2}$,^33^ where F(·,·;ρ) is the standardized bivariate normal distribution function with correlation ρ, it follows that ${\hat{\sigma }}_{R:\mathrm{OR}}^{2}-F_{\mathrm{BVN}}(x_{1},x_{2};x_{3})+\Phi (x_{1})\Phi (x_{2})$ is an increasing function of $x_{3}$ and hence $x_{3}$ can be easily determined numerically. Numerical solutions for $x_{4},x_{5},x_{6}$, and $x_{7}$ can be similarly determined in steps 3 and 6.
**Step 3**. Solve for $x_{4}$, using the values for $x_{1}$ and $x_{2}$ obtained in step 1:
x4=max[x3,{0≤x4<1:σ^TR:OR2+σ^R:OR2−.5∑i=12{FBVN(xi,xi;x4)−[Φ(xi)]2}=0}]
**Step 4**. Solve for b using one of the following *b_method* options. The resulting value of b is used for the remaining steps.
*b_method* = unspecified: Solve for b, using the values for $x_{1},x_{2}$, and $x_{4}$ obtained in steps 1 and 3:
b={b>0:σ^ε:OR2−12∑i=12∑m=14cmFBVN(xi,xi;ρm(1−x4)+x4)=0}
where $\rho_{1}=1,\;\rho_{2}=\frac{1}{1+b^{-2}},\;\rho_{3}=\frac{1}{1+b^{2}},\;\rho_{4}=0$. With this option there can be 0, 1, or 2 possible solutions for b. The algorithm returns the largest solution such that 0.001≤b≤1 if it exists; otherwise, it returns the smallest solution such that 1≤b≤4 if it exists, or a missing value if it does not exist.
*b_method* = specified: Use the specified value of b.
*b_method* = mean_to_sigma: Solve for the value of b that corresponds to a specified mean-to-sigma ratio and the minimum of the specified values for the expected test 1 and test 2 AUCs. (See Sec. B.2 for details.)
**Step 5**. Compute OR covariance estimates to be used in step 6.
(a) If *b_method* = unspecified was used in step 4, compute
Cov^i=r^iσ^ε:OR2,i=1,2,3.
(b) If one of the other two methods was used in step 4, then using the computed value of b and the inputted correlations ${\hat{r}}_{1},{\hat{r}}_{2}$ and ${\hat{r}}_{3}$, compute a new value for the OR error variance, given by ${\tilde{\sigma }}_{\varepsilon :\mathrm{OR}}^{2}=\frac{1}{2}\sum_{i=1}^{2}\sum_{m=1}^{4}c_{m}F_{\mathrm{BVN}}(x_{i},x_{i};\rho_{m}(1-x_{4})+x_{4})$, where $\rho_{1}=1,\;\rho_{2}=\frac{1}{1+b^{-2}},\;\rho_{3}=\frac{1}{1+b^{2}},\;\rho_{4}=0.$ Then compute
Cov^i=r^iσ˜ε:OR2,i=1,2,3.
**Step 6**. Solve for $x_{5},x_{6,}$ and $x_{7}$, using the following equations and the values for $x_{1},x_{2},x_{4}$, b and ${\hat{\mathrm{Cov}}}_{i},i=1,2,3$, obtained in steps 1, 3, and 5:
x5={0≤x5<1:Cov^3−∑m=14cmFBVN(x1,x2;ρm(1−x4))=0}
where $\rho_{1}=x_{5},\rho_{2}=\frac{x_{5}}{1+b^{-2}},\rho_{3}=\frac{x_{5}}{1+b^{2}},\rho_{4}=0$
x6=max[x5,{0≤x6<1:Cov^2−12∑i=12∑m=14cmFBVN(xi,xi;ρm(1−x4))=0}]
where $\rho_{1}=x_{6},\;\rho_{2}=\frac{x_{6}}{1+b^{-2}},\;\rho_{3}=\frac{x_{6}}{1+b^{2}},\;\rho_{4}=0$
x7=max[x5,{0≤x7<1:Cov^1−∑m=14cmFBVN(x1,x2;ρm(1−x4)+x3)=0}]
where $\rho_{1}=x_{7},\;\rho_{2}=\frac{x_{7}}{1+b^{-2}},\;\rho_{3}=\frac{x_{7}}{\left(1+b^{2}\right)},\;\rho_{4}=0$
**Step 7**. Solve for the estimated RMH parameter values as functions of the estimated alternative RMH parameter values using the mapping given in Table 7c.

Notes: ${\hat{\theta }}_{1}$ and ${\hat{\theta }}_{2}$ denote specified values of the reader-averaged performance empirical AUCs for tests 1 and 2, respectively; ${\hat{\sigma }}_{R:\mathrm{OR}}^{2},{\hat{\sigma }}_{TR:\mathrm{OR}}^{2}$, and ${\hat{\sigma }}_{\varepsilon :\mathrm{OR}}^{2}$ denote specified values of the corresponding OR parameters, and ${\hat{r}}_{1},{\hat{r}}_{2}$, and ${\hat{r}}_{3}$ denote specified values for the OR correlations defined by $r_{i}={\mathrm{Cov}}_{i:\mathrm{OR}}/\sigma_{\varepsilon :\mathrm{OR}}^{2}$. These specified values can be computed from real data or conjectured. $F_{\mathrm{BVN}}(.,.;\rho )$ is the standardized bivariate normal distribution function with correlation ρ. Note that constraints Eq. (23) in Table 7 have been incorporated into the preceding steps.

**Table 11:** Troubleshooting the OR-to-RMH algorithm when missing parameter values result.

Alternative RMH parameter	When the parameter to the left is the first parameter in the column to have a missing value, try the following corrective action:
$x_{1}$	NA (should be no problem)
$x_{2}$	NA (should be no problem)
$x_{3}$	Reduce the value of $\sigma_{R}^{2}$
$x_{4}$	Reduce the value of $\sigma_{TR}^{2}$
b	If using b_method = unspecified, there are two possible solutions:
	(a) Change (reduce or increase) the value of $\sigma_{\varepsilon ;\mathrm{OR}}^{2}$
	(b) Use one of the other two b_method options, which should always work
$x_{5}$	Reduce the value of $r_{3}$
$x_{6}$	Reduce the value of $r_{2}$
$x_{7}$	Reduce the value of $r_{1}$

The proposed algorithm is presented in Table 10. Steps 1 to 6 replace the OR parameters in Table 8 by specified values and then solve for the corresponding alternative RMH parameter values. Note that these steps incorporate the alternative parameter constraints given in Table 7(b). Using Table 7(c) mappings, step 7 computes the final RMH parameter estimates as functions of the estimated alternative RMH parameter values.

From Table 9, it follows that for each of the alternative parameters other than b, there can be only one solution. It then follows from Table 8(a) that there can be only one solution for the RMH parameters other than b. Hence, if there is more than one solution, they differ only in their b values.

Sometimes there is not an exact or approximate solution and the OR-to-RMH algorithm returns missing values. When this happens, changing the values of the inputted OR parameters or changing the b_method option will generally result in a solution, as discussed in Sec. 3.1.3. The algorithm solves for the alternative RMH parameters in the following order: $x_{1},x_{2},x_{3},x_{4},b,x_{5},x_{6}$, and $x_{7}$. Because the parameters may require estimates of preceding but not subsequent parameters, all parameters following a parameter with no solution are assigned a missing value by the algorithm. Table 11 describes the appropriate correction action that will produce a solution for the OR-to-RMH algorithm, according to which is the first RMH parameter to not have a solution.

## 8 Appendix B: Mean-to-Sigma Details

### 8.1 B.1 Computation of the Probability of a Noticeably Improper ROC Curve

For the RMH model, the mean-to-sigma ratio varies across readers. Letting $q_{ij}$ denote the mean-to-sigma ratio for test i and reader j, Hillis^3^ shows the RMH model implies that

$$q_{ij}∼N[q_{i},\frac{2(\sigma_{R}^{2}+\sigma_{TR}^{2})}{{\left(b^{-1}-1\right)}^{2}}],\;i=1,2,$$

where

$$q_{i}=\frac{\delta_{i}}{\left(b^{-1}-1\right)}.$$

It follows for test i that the probability that a reader’s ROC curve is noticeably improper (i.e., the absolute value of the mean-to-sigma ratio is less than 2, as discussed in Sec. 3.2.2) is given as

$$\mathrm{Pr}(|q_{ij}|<2)=\Phi (\frac{2-q_{i}}{\overset{˜}{\sigma }})-\Phi (\frac{-2-q_{i}}{\overset{˜}{\sigma }}),$$

where

$$\overset{˜}{\sigma }=\sqrt{\frac{2(\sigma_{R}^{2}+\sigma_{TR}^{2})}{{\left(b^{-1}-1\right)}^{2}}}.$$

### 8.2 B.2 Derivation of b in Step 4 in Table 10 when b_method = mean_to_sigma

Without loss of generality, we assume that test 1 has the lower OR AUC input; i.e., $\mu_{1:\mathrm{OR}}=\min(\mu_{1:\mathrm{OR}},\mu_{2:\mathrm{OR}})$. Let ${\hat{\theta }}_{1j}$ denote the empirical AUC estimate for a randomly selected RMH reader j reading a random RMH sample of ratings for test 1. Given the solution values of $x_{1},x_{2},x_{3}$, and $x_{4}$ from steps 1 to 4 in Table 6, we want to solve for b such that $E({\hat{\theta }}_{1j})=\mu_{1;\mathrm{OR}}$ and $\text{median}(q_{j})=q$, where $q_{j}$ is the mean-to-sigma ratio for reader j.

Recall that for test 1, the median separation between the latent normal and abnormal distributions for test 1 across readers is equal to $\delta_{1}$. It follows that the median mean-to-sigma ratio is given by

$$q=\frac{\delta_{1}}{b^{-1}-1},$$

and hence

$$\delta_{1}=q(b^{-1}-1).$$ (24)

From Table 6, we can write

$$\Phi^{-1}(\mu_{1:\mathrm{OR}})=\delta_{1}/\sqrt{V}\;=\frac{\delta_{1}}{\sqrt{1+b^{-2}+2(\sigma_{R}^{2}+\sigma_{TR}^{2})}}.$$

Using the relationship $1-x_{4}=[1+b^{-2}]/V$ from Table 7, it follows that

$$\Phi^{-1}(\mu_{1:\mathrm{OR}})=\frac{\delta_{1}}{\sqrt{1+b^{-2}[\frac{1}{1-x_{4}}]}}.$$ (25)

Substituting expression Eq. (24) for $\delta_{1}$ into Eq. (25) yields

$$\Phi^{-1}(\mu_{1:\mathrm{OR}})=\frac{r(b^{-1}-1)}{\sqrt{\left(1+b^{-2})[\frac{1}{1-x_{4}}\right]}},$$

which implies

$${\left[\Phi^{-1}(\mu_{1:\mathrm{OR}})\right]}^{2}=\frac{r^{2}b^{-2}-2r^{2}b^{-1}+r^{2}}{\left(1+b^{-2})[\frac{1}{1-x_{4}}\right]},$$

or equivalently

$${\left[\Phi^{-1}(\mu_{1:\mathrm{OR}})\right]}^{2}(1+b^{-2})[\frac{1}{1-x_{4}}]-r^{2}b^{-2}+2r^{2}b^{-1}-r^{2}=0.$$ (26)

Collecting terms in Eq. (26) results in a quadratic equation in $b^{-1}$:

$$b^{-2}\{{\left[\Phi^{-1}(\mu_{1:\mathrm{OR}})\right]}^{2}[\frac{1}{1-x_{4}}]-r^{2}\}+b^{-1}(2r^{2})+[\Phi^{-1}(\mu_{1:\mathrm{OR}})][\frac{1}{1-x_{4}}]-r^{2}=0.$$

Solving for $b^{-1}$ using the quadratic equation formula yields

$$b^{-1}=\frac{-b_{1}\pm \sqrt{b_{1}^{2}-4a_{1}c_{3}}}{2a_{1}},$$

where

$$a_{1}=[\Phi^{-1}(\mu_{1:\mathrm{OR}}){]}^{2}[\frac{1}{1-x_{4}}]-r^{2},\;b_{1}=2r^{2},\;c_{3}=[\Phi^{-1}(\mu_{1:\mathrm{OR}}){]}^{2}[\frac{1}{1-x_{4}}]-r^{2}.$$

## 9 Appendix C: Commands and Output for Tables from Applying the OR_to_RMH and RMH_to_OR R Functions

This appendix includes the R commands and resulting output that were used to produce the content of Fig. 4 and Tables 3–5. Note that both the RMH_to_OR and RMH_to_OR functions return values for *mean_to_sig1, mean_to_sig2, mean_sig1_025*, and *mean_sig2_025*; these are not RMH-model or OR-model parameters but rather are parameters describing the distributions of the true reader AUC values.

### 9.1 C.1 R Commands and Output Corresponding to Fig. 4

#### 9.1.1 C.1.1 Computation of RMH values by applying OR-to-RMH algorithm to VanDyke original OR values

> VanDyke_OR_orig_values <- data.frame(n0 = 69, n1 = 45, AUC1 = 0.897,

+ AUC2 = 0.941, var_R = 0.00154, var_TR = 0.000208,

+ error_var = 0.000788, corr1 = 0.433, + corr2 = 0.430, corr3 = 0.299)

> RM_values <- OR_to_RMH(VanDyke_OR_orig_values)

> print(RM_values)

n0 n1 delta1 delta2 var_R var_TR var_C var_TC

1 69 45 2.392224 2.957029 0.1223413 0.005180485 0.4716964 0.1222262

var_RC var_error b b_method mean_to_sig1 mean_to_sig2

1 0.1091448 0.2969327 0.656081 unspecified 4.563553 5.64101

Pr1_improper Pr2_improper

1 0.003896242 7.862956e-05

#### 9.1.2 C.1.2 Computation of OR true values by applying RMH-to-OR algorithm to RMH values

> OR_true_values <- RMH_to_OR(RM_values)

> print(OR_true_values)

n0 n1 AUC1 AUC2 var_R var_TR error_var cov1

1 69 45 0.897 0.941 0.00154 0.000208 0.0007880002 0.0003412041

cov2 cov3 corr1 corr2 corr3 b mean_to_sig1

1 0.0003388401 0.0002356121 0.433 0.43 0.299 0.656081 4.563553

mean_to_sig2 Pr1_improper Pr2_improper

1 5.64101 0.003896242 7.862956e-05

### 9.2 C.2 R Commands and Output Corresponding to Table 3

> # Create data frame with 5 rows, with row 1 same as RM_values in Table 3a

> # and rows 2-5 changed slightly.

> VanDyke_OR_orig_values <- data.frame(n0 = 69, n1 = 45, AUC1 = 0.897,

+ AUC2 = 0.941, var_R = 0.00154, var_TR = 0.000208, error_var = 0.000788, + corr1 = 0.433, corr2 = 0.430, corr3 = 0.299)

> RM_values <- OR_to_RMH(VanDyke_OR_orig_values)

> RM_Table4 <- RM_values[c(1,1,1,1,1),] #creates data frame with 5 rows, each = RM_values

> RM_Table34[2,c("n0","n1")] <- c(138, 90)

> RM_Table34[3,c("n0","n1")] <- c(45, 69)

> RM_Table34[4,c("delta1","delta2")] <- c(2.6452, 2.6452)

> RM_Table34[5,c("delta1","delta2")] <- c(1.2759, 1.2759)

> print(RM_Table3)

n0 n1 delta1 delta2 var_R var_TR var_C

1 69 45 2.392224 2.957029 0.1223413 0.005180485 0.4716964

1.1 138 90 2.392224 2.957029 0.1223413 0.005180485 0.4716964

1.2  45 69 2.392224 2.957029 0.1223413 0.005180485 0.4716964

1.3  69 45 2.645200 2.645200 0.1223413 0.005180485 0.4716964

1.4  69 45 1.275900 1.275900 0.1223413 0.005180485 0.4716964

var_TC var_RC var_error b b_method mean_to_sig1

1 0.1222262 0.1091448 0.2969327 0.656081 unspecified 4.563553

1.1 0.1222262 0.1091448 0.2969327 0.656081 unspecified 4.563553

1.2 0.1222262 0.1091448 0.2969327 0.656081 unspecified 4.563553

1.3 0.1222262 0.1091448 0.2969327 0.656081 unspecified 4.563553

1.4 0.1222262 0.1091448 0.2969327 0.656081 unspecified 4.563553

mean_to_sig2 Pr1_improper Pr2_improper

1 5.64101 0.003896242 7.862956e-05

1.1 5.64101 0.003896242 7.862956e-05

1.2 5.64101 0.003896242 7.862956e-05

1.3 5.64101 0.003896242 7.862956e-05

1.4 5.64101 0.003896242 7.862956e-05

> OR_values_Table3 <- RMH_to_OR(RM_Table3)

> print(OR_values_Table3)

n0 n1 AUC1 AUC2 var_R var_TR error_var

1 69 45 0.8970000 0.9410000 0.001540000 2.080000e-04 0.0007880002

1.1 138 90 0.8970000 0.9410000 0.001540000 2.080000e-04 0.0003912576

1.2 45 69 0.8970000 0.9410000 0.001540000 2.080000e-04 0.0006344427

1.3 69 45 0.9190000 0.9190000 0.001644069 7.426773e-05 0.0007890063

1.4 69 45 0.7500034 0.7500034 0.007014410 3.019443e-04 0.0023458109

cov1 cov2 cov3 corr1 corr2

1 0.0003412041 0.0003388401 0.0002356121 0.4330000 0.4300000

1.1 0.0001703301 0.0001691406 0.0001176498 0.4353401 0.4322997

1.2 0.0002800701 0.0002778178 0.0001940871 0.4414426 0.4378927

1.3 0.0003644012 0.0003363961 0.0002513892 0.4618483 0.4263542

1.4 0.0012240655 0.0012083227 0.0009406161 0.5218091 0.5150981

corr3 b mean_to_sig1 mean_to_sig2 Pr1_improper

1 0.2990000 0.656081 4.563553 5.641010 0.003896242

1.1 0.3006966 0.656081 4.563553 5.641010 0.003896242

1.2 0.3059174 0.656081 4.563553 5.641010 0.003896242

1.3 0.3186150 0.656081 5.046146 5.046146 0.000783834

1.4 0.4009769 0.656081 2.433985 2.433985 0.326185605

Pr2_improper

1 7.862956e-05

1.1 7.862956e-05

1.2 7.862956e-05

1.3 7.838340e-04

1.4 3.261856e-01

### 9.3 C.3 R Commands and Output Corresponding to Table 4

> VanDyke_OR_orig_values <- data.frame(n0 = 69, n1 = 45, AUC1 = 0.897,

+ AUC2 = 0.941, var_R = 0.00154, var_TR = 0.000208, var_error = 0.000788,

+ corr1 = 0.433, corr2 = 0.430, corr3 = 0.299)

> Table4_OR1 <- VanDyke_OR_orig_values[c(1,1,1),] #creates data frame with 3 rows,

> # each the same as VanDyke_OR_orig_values

> Table4_OR2 <- data.frame(b_method=c("unspecified", "mean_to_sigma","specified"),

+ b_input = c(NA,NA,1), mean_sig_input = c(NA,5.2,NA))

> Table4_OR <- cbind(Table5_OR1, Table5_OR2)

> print("Original OR parameter values")

[1] "Original OR parameter values"

> print(Table4_OR)

n0 n1 AUC1 AUC2 var_R var_TR var_error corr1 corr2 corr3

1 69 45 0.897 0.941 0.00154 0.000208 0.000788 0.433 0.43 0.299

1.1 69 45 0.897 0.941 0.00154 0.000208 0.000788* 0.433 0.43 0.299

1.2 69 45 0.897 0.941 0.00154 0.000208 0.000788* 0.433 0.43 0.299

b_method b_input mean_sig_input

1 unspecified NA NA

1.1 mean_to_sigma NA 5.2

1.2 specified 1 NA

*Note that with *mean_to_sigma* = mean_to_sigma or specified it is not necessary to specify a value for *var_error*, or the value can be NA

> Table4_RMH <- OR_to_RMH(Table4_OR)

> print("Table 4 RMH parameter values")

[1] "Table 4 RMH parameter values"

> print(Table4_RM)

n0 n1 delta1 delta2 var_R var_TR var_C

1 69 45 2.392224 2.957029 0.12234134 0.005180485 0.4716964

1.1 69 45 2.303940 2.847902 0.11347812 0.004805176 0.4674676

1.2 69 45 1.855834 2.293997 0.07362882 0.003117776 0.4498198

var_TC var_RC var_error b b_method

1 0.1222262 0.1091448 0.2969327 0.6560810 unspecified

1.1 0.1220955 0.1089342 0.3015027 0.6929693 mean_to_sigma

1.2 0.1215947 0.1080172 0.3205683 1.0000000 specified

mean_to_sig1 mean_to_sig2 Pr1_improper Pr2_improper

1 4.563553 5.641010 0.003896242 7.862956e-05

1.1 5.200000 6.427723 0.001778344 2.748745e-05

1.2 Inf Inf 0.000000000 0.000000e+00

> Table5_true_values <- RM_to_OR(Table4_RM)

> print("Table 4 True OR values")

[1] "Table 4 True OR values"

> print(Table4_true_values)

n0 n1 AUC1 AUC2 var_R var_TR var_error cov1

1 69 45 0.897 0.941 0.00154 0.000208 0.0007880002 0.0003412041

1.1 69 45 0.897 0.941 0.00154 0.000208 0.0007664249 0.0003318620

1.2 69 45 0.897 0.941 0.00154 0.000208 0.0006584975 0.0002851294

cov2 cov3 corr1 corr2 corr3 b

1 0.0003388401 0.0002356121 0.433 0.43 0.299 0.6560810

1.1 0.0003295627 0.0002291610 0.433 0.43 0.299 0.6929693

1.2 0.0002831539 0.0001968908 0.433 0.43 0.299 1.0000000

mean_to_sig1 mean_to_sig2 Pr1_improper Pr2_improper

1 4.563553 5.641010 0.003896242 7.862956e-05

1.1 5.200000 6.427723 0.001778344 2.748745e-05

1.2 Inf Inf 0.000000000 0.000000e+00

### 9.4 C.4 R Commands and Output Corresponding to Table 5

#### 9.4.1 C.4.1 Table 5(a) code ($\sigma_{R:\mathrm{OR}}^{2}$ changed from 0.00154 to 0.154)

> VanDyke_OR_altered_values_a <- data.frame(n0 = 69, n1 = 45, AUC1 = 0.897,

+ AUC2 = 0.941, var_R = 0.154, var_TR = 0.000208, var_error = 0.000788,

+ corr1 = 0.433, corr2 = 0.430, corr3 = 0.299)

> RM_values = OR_to_RM(VanDyke_OR_altered_values_a)

*Warning message: In OR_to_RM.default(n0 = 69, n1 = 45, AUC1 = 0.897, AUC2 = 0.941, : Conversion failed. Try reducing the value of var_R.*

> print(RM_values,all=T)

n0 n1 delta1 delta2 var_R var_TR var_C var_TC var_RC var_error b

1 69 45 NA NA NA NA NA NA NA NA NA

b_method mean_to_sig1 mean_to_sig2 Pr1_improper Pr2_improper

1 unspecified NA NA NA NA

x1 x2 x3 x4 x5 x6 x7

1 1.264641 1.563224 NA NA NA NA NA

#### 9.4.2 C.4.2 Table 5(b) code ($\sigma_{TR:\mathrm{OR}}^{2}$ changed from 0.00028 to 0.28)

> VanDyke_OR_altered_values_b <- data.frame(n0 = 69, n1 = 45, AUC1 = 0.897,

+ AUC2 = 0.941, var_R = 0.00154, var_TR = 0.208, var_error = 0.000788,

+ corr1 = 0.433, corr2 = 0.430, corr3 = 0.299)

> RM_values <- OR_to_RM(VanDyke_OR_altered_values_b)

*Warning message: In OR_to_RM.default(n0 = 69, n1 = 45, AUC1 = 0.897, AUC2 = 0.941, : Conversion failed. Try reducing the value of var_TR.*

> print(RM_values,all=T)

n0 n1 delta1 delta2 var_R var_TR var_C var_TC var_RC var_error b

1 69 45 NA NA NA NA NA NA NA NA NA

b_method mean_to_sig1 mean_to_sig2 Pr1_improper Pr2_improper

1 unspecified NA NA NA NA

x1 x2 x3 x4 x5 x6 x7

1 1.264641 1.563224 0.06838082 NA NA NA NA

#### 9.4.3 C.4.3 Table 5(c) code ($\sigma_{\varepsilon :\mathrm{OR}}^{2}$ changed from 0.000788 to 0.00788)

> VanDyke_OR_altered_values_c <- data.frame(n0 = 69, n1 = 45, AUC1 = 0.897,

+ AUC2 = 0.941, var_R = 0.00154, var_TR = 0.000208, var_error = 0.00788,

+ corr1 = 0.433, corr2 = 0.430, corr3 = 0.299)

> RM_values <- OR_to_RM(VanDyke_OR_altered_values_c)

*Warning message: In OR_to_RM.default (n0 = 69, n1 = 45, AUC1 = 0.897, AUC2 = 0.941, : Conversion failed. If using b_method = "unspecified," there are two possible solutions: (a) Try changing (reduce or increase) the value of var_error.( b) Try using one of the other two b_method options, which should always work.*

> print(RM_values,all=T)

n0 n1 delta1 delta2 var_R var_TR var_C var_TC var_RC var_error b

1 69 45 NA NA NA NA NA NA NA NA NA

b_method mean_to_sig1 mean_to_sig2 Pr1_improper Pr2_improper

1 unspecified NA NA NA NA

x1 x2 x3 x4 x5 x6 x7

1 1.264641 1.563224 0.06838082 0.07127637 NA NA NA
